# Hydrophilic and Hydrophobic Nanostructured Copper Surfaces for Efficient Pool Boiling Heat Transfer with Water, Water/Butanol Mixtures and Novec 649

**DOI:** 10.3390/nano11123216

**Published:** 2021-11-26

**Authors:** Matic Može, Viktor Vajc, Matevž Zupančič, Iztok Golobič

**Affiliations:** 1 Faculty of Mechanical Engineering, University of Ljubljana, Aškerčeva 6, 1000 Ljubljana, Slovenia; iztok.golobic@fs.uni-lj.si; 2 Faculty of Mechanical Engineering, Czech Technical University in Prague, Technická 4, 160 00 Prague 6, Czech Republic; viktor.vajc@fs.cvut.cz

**Keywords:** copper nanostructures, surface engineering, pool boiling, nucleate boiling, enhanced heat transfer, self-rewetting fluids, Novec 649

## Abstract

Increasing heat dissipation requirements of small and miniature devices demands advanced cooling methods, such as application of immersion cooling via boiling heat transfer. In this study, functionalized copper surfaces for enhanced heat transfer are developed and evaluated. Samples are functionalized using a chemical oxidation treatment with subsequent hydrophobization of selected surfaces with a fluorinated silane. Pool boiling tests with water, water/1-butanol mixture with self-rewetting properties and a novel dielectric fluid with low GWP (Novec™ 649) are conducted to evaluate the boiling performance of individual surfaces. The results show that hydrophobized functionalized surfaces covered by microcavities with diameters between 40 nm and 2 µm exhibit increased heat transfer coefficient (HTC; enhancements up to 120%) and critical heat flux (CHF; enhancements up to 64%) values in comparison with the untreated reference surface, complemented by favorable fabrication repeatability. Positive surface stability is observed in contact with water, while both the self-rewetting fluids and Novec™ 649 gradually degrade the boiling performance and in some cases also the surface itself. The use of water/1-butanol mixtures in particular results in surface chemistry and morphology changes, as observed using SEM imaging and Raman spectroscopy. This seems to be neglected in the available literature and should be focused on in further studies.

## 1. Introduction

Boiling heat transfer is a process often employed in various industrial apparatuses, such as heat exchangers, distillation columns, cooling systems, nuclear reactors, etc. It is characterized by intense heat transfer at a relatively small temperature difference and is therefore seeing increased use in heat dissipation applications involving electronic components.

### 1.1. Basics of Boiling Heat Transfer

**Figure 1:**
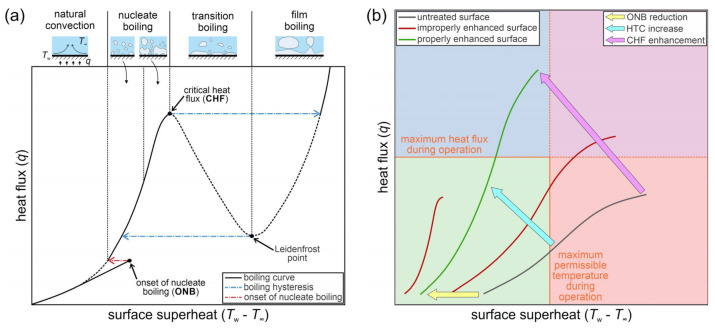
Schematic depiction of a typical pool boiling curve together with its different regions (**a**) and the key optimization factors for boiling performance enhancement (**b**) [3].

Pool boiling performance can be described using a boiling curve, as shown schematically in Figure 1a. At low heat fluxes and low surface superheat (i.e., temperature difference between the surface and the boiling fluid), all of the heat is dissipated through natural convection. When the thermal load is sufficiently high, onset of nucleate boiling (ONB) occurs, vapor bubbles start forming on the surface and nucleate boiling regime is initiated. The heat transfer coefficient (HTC) is characterized as the ratio of the heat flux and the surface superheat. The nucleate boiling regime tends to provide the highest HTC values observed during pool boiling and is therefore the most suitable for engineering applications. As the heat flux increases in the nucleate boiling regime, the density of active nucleation sites producing bubbles also increases. When merging of the bubbles on and above the boiling surface is so intense that an insulative vapor film forms, the critical heat flux (CHF) is reached, and the boiling process transitions toward film boiling, which is accompanied by significant reduction of the HTC.

Some applications require enhancement of boiling performance, as increasingly smaller devices with higher power are developed [1,2]. The main heat transfer parameters that characterize boiling (ONB, HTC and CHF) are focused on during boiling enhancement research. Boiling performance enhancement can be shown through a boiling curve shift as depicted in Figure 1b. Most studies focus on ONB reduction through the shift of the curve toward lower superheat values, increased HTC values through higher steepness of the curve and higher CHF values. Boiling performance can be intensified either by modification of surface parameters or by modification of properties of the boiling liquid (surface tension, latent heat of vaporization, viscosity and others) [3,4]. The interaction of the surface with the boiling liquid is also important in influencing boiling behavior. Most research is focused on developing functionalized surfaces for boiling enhancement through surface engineering methods, by changing the surface morphology and their interaction with the boiling fluid [3,5,6,7]. Boiling surfaces tailored toward enhancement of boiling performance are highly required in industrial applications [8,9]. The performance of boiling surfaces depends on a variety of interconnected factors, such as roughness, topology, porosity, wettability, wickability and others [10,11,12,13] and might vary in time due to surface aging, fouling, oxidation or contamination [3,11,14,15,16,17].

### 1.2. Chemical Oxidation of Copper Surfaces and Their Structure

Surfaces made of copper are the most often studied in literature dealing with pool boiling. However, boiling performance of copper often varies in time due to its reactivity and morphological changes, especially in humid conditions [18]. Improvement, as well as decrease in boiling performance of copper surfaces due to boiling-induced aging, was reported in the literature [19]. Bare copper surfaces are characterized by rather poor wettability, which can negatively impact some aspects of boiling performance, such as the CHF value [20]. Static contact angles for bare copper are often greater than 70° [18], while copper surfaces covered with passivating oxide layers typically exhibit increased wettability [16].

Controlled chemical oxidation is a method of surface treatment studied by various researchers [18,21,22,23,24,25,26], which affects surface energy, as well as morphology of metallic surfaces, and can significantly affect boiling performance and stability of copper surfaces [18,20]. Oxidized surfaces can then be further functionalized [27]. Copper is especially suitable for micro- and nanostructuring using chemical oxidation, as oxide layers do not substantially increase its thermal resistance and can improve boiling performance [14,21,28,29,30,31]. Synergy between the micro- and nanostructure can create liquid supply pathways, strengthen the interactions between boiling liquid and surface and improve the stability of microcavities [12,15,31,32].

One of the simplest methods of chemical oxidation of various metal surfaces is by submerging them into an alkaline solution [25]. This method requires neither specialized equipment nor high temperatures [33]. It can produce uniform oxide layers of high roughness without causing any thermal stresses associated with thermal oxidation processes [18]. The resulting surface structure (in terms of its shape, complexity and thickness) is highly dependent on the composition and concentration of the used alkaline solution [18,25], temperature [25,26,34] and reaction time [18,34]. Longer reaction times, higher temperatures and increased pH accelerate oxidation [26]. For maximum HTC enhancement, the size of structures is important and should correspond to the size required for effective nucleation for a given boiling liquid [35].

### 1.3. Surface Hydrophobization

Surface wettability describes the behavior of the surface when in contact with a specific liquid. Wettability is typically characterized by the contact angle at the foot of a droplet deposited onto the surface. When a liquid spreads easily across the surface, it will form a low contact angle, and the surface will be considered “philic” to that liquid (hydrophilic in case of water). On the contrary, when spreading of the liquid is not observed and the liquid remains in partly spherical shape with a contact angle greater than 90°, the surface is deemed “phobic” to that liquid (hydrophobic in case of water). Overall, surface wettability is important in influencing boiling heat transfer. Wettability is mostly affected by free surface energy (i.e., surface chemistry), by roughness [8,34] of the surface, by its microstructure and nanostructure [3,18,36], and it is also a precursor for surface wickability, which is reported to significantly affect boiling performance [11,14,37]. It is mostly accepted that highly wettable (philic) surfaces will grant higher CHF values compared with their phobic counterparts, but with the drawback of higher surface superheat values [38,39]. Nevertheless, properly degassed (super)hydrophobic surfaces (i.e., ones without entrapped non-condensable gasses) have recently been reported to be capable of significantly enhancing the HTC and lowering the ONB value without the penalty of low CHF values [40,41,42].

Boiling surfaces can be further functionalized with a hydrophobizing agent to enhance boiling performance. Hydrophobized surfaces can also possess several interesting properties, such as self-cleaning ability, resistance to corrosion and fouling, decreased frost growth rates, reduced drag and others [27,43]. However, hydrophobization also results in the weakening or suppression of surface rewetting, leading to larger bubbles, longer nucleation frequencies, more intense coalescence or a decrease in CHF due to suppression of capillary wicking [24,36].

### 1.4. Self-Rewetting Fluids

While surface modification is the most often applied technique for boiling enhancement, the properties of the boiling fluid also have an important role in phase-change heat transfer. The boiling of binary or multi-component mixtures is significantly more complex than that of single-component pure fluids. Many existing publications show that even trace amounts of one of the two pure fluids in the mixture lead to results that cannot be predicted by simple interpolation between the pure mixture components [44,45,46,47,48]. This dependence is strongly non-linear, and there are several possible reasons for the non-linear decrease in the HTC compared with pure water or pure butanol [44,49,50,51]. Firstly, there is a local rise of the saturation and surface temperature due to preferred evaporation of the more volatile components, accompanied by the appearance of concentration gradients. Furthermore, limited mass diffusion of the more volatile components toward the phase boundary of the bubbles decreases heat transfer intensity, and the effects of the mixture’s physical properties influence the nucleation phenomena.

Although negligible in the boiling of single-component fluids [52], Marangoni flow significantly affects the boiling performance in dilute mixtures of alcohols (with four and more carbon atoms) in water [53,54]. These mixtures were named self-rewetting fluids (SRFs), as solutal Marangoni flow is expected to be further enhanced by thermal Marangoni flow, and the inflow of colder liquid spontaneously rewets hot places during boiling due to surface tension gradients induced by temperature inhomogeneities inside the boiling liquid [9,52,54].

Although the expected increase in CHF due to the inflow of cold liquid toward the boiling surface was confirmed for confined spaces and restricted boiling surfaces (wires, microchannels, etc.) [55,56], the results for flat surfaces are ambiguous and unpredictable [55,57,58]. Research on the pool boiling of SRFs on modified surfaces is scarce. Hu et al. [59] investigated the boiling of mixtures of heptanol and water on flat titanium surfaces with titanium dioxide nanotubes prepared via anodic oxidation and reported significant enhancement of HTC and CHF. Zhou et al. [60] experimented with the boiling of 1-butanol/water mixtures on a flat copper surface bonded with copper foam and achieved minor enhancement of heat transfer using their densest foam. Sankaran et al. [9] studied the boiling of 1-heptanol/water mixtures on flat copper surfaces with polymer nanostructures. Although a small HTC improvement was achieved, CHF was lower compared with the boiling of pure water. Sahu et al. [54] boiled a mixture of 1-heptanol and water on a flat copper surface covered with copper-plated polymer nanofibers and recorded an enhancement of heat transfer.

### 1.5. Boiling Heat Transfer with Dielectric Fluids

Several applications that rely on boiling heat transfer for heat dissipation purposes require the working fluid to be dielectric and have a suitably low boiling point compared with the maximal permissible temperatures of the cooled components. Consequently, so-called dielectric fluids with good thermal and chemical stability and material compatibility are typically used. While dielectric fluids are suitable for applications such as direct-immersion cooling of electronic components, they are also typically characterized by poor thermophysical properties. As a consequence, the achievable cooling performance is significantly lower compared with pure water, as both the HTC and the CHF tend to be one order of magnitude lower for common dielectric fluids with respect to the boiling of pure water under the same operating conditions [61,62].

So far, only a few studies were performed using environmentally friendly dielectric fluid 3M™ Novec™ 649, which has an ultra-low global warming potential (GWP) in comparison with the well-known dielectric 3M™ Fluorinert™ FC-72, which has similar thermophysical properties [63,64]. Kaniowski et al. [65,66,67] published several papers on boiling heat transfer enhancement using Novec™ 649 as the working fluid. They obtained HTCs up to approx. 9 kW m^−2^ K^−1^ using open microchannels. Recently, Pastuszko et al. [68] investigated the use of deep microchannels for boiling enhancement and found that the best results for Novec™ 649 were obtained at a depth of 10 mm with the highest recorded HTCs exceeding 25 kW m^−2^ K^−1^. Ghaffari et al. [69] measured the CHF of Novec™ 649 to be 195 kW m^−^^2^ on bare copper surface under pool boiling conditions. Cao et al. [70] enhanced the pool boiling heat transfer performance of Novec™ 649 by coating the boiling surface with either micro- or nanoparticles. In another study [71], the same team also developed microporous surfaces capable of boiling performance enhancement in combination with Novec™ 649. These functionalized surfaces had high roughness values and provided an abundance of active nucleation sites, which helped increase the HTC and the CHF by 600% and 55%, respectively. Hsu et al. [72] studied the enhancement of pool boiling of Novec™ 649 using silicon nanowire/nanopillar one- and two-tier structures. The highest CHF of 235 kW m^−2^ was recorded alongside a maximal HTC of 23.6 kW m^−2^ K^−1^. Even though the existing studies already showed that the boiling performance of Novec™ 649 can be enhanced via surface functionalization, the use of low-surface-energy coating in combination with modified surface morphology has not yet been studied and remains a promising topic to explore.

### 1.6. Motivation of This Work

Only a few works investigated the boiling performance of functionalized copper surfaces with modified micro- and nanostructure and additionally altered wettability. Furthermore, investigations of the performance of enhanced surfaces in combination with more than one boiling fluid are rare. The possibility of using several methods of boiling heat transfer enhancement at once is also largely unexplored. Finally, fabrication repeatability, surface stability and the aging of developed surfaces under boiling conditions are absent in most studies. Therefore, we aim to comprehensively explore: (i) the possibility of using low-surface-energy coating to further enhance boiling on surfaces with modified morphology; (ii) the effects of using self-rewetting fluids in combination with developed surfaces to further increase their boiling performance; and to (iii) evaluate the performance of developed surfaces using a dielectric fluid with a significantly lower surface tension than water and different wetting behavior.

For this purpose, the study investigates the pool boiling performance of functionalized copper surfaces in combination with pure water, self-rewetting mixtures of 1-butanol and water and a dielectric fluid Novec™ 649. Surfaces are nanostructured through controlled chemical oxidation in alkaline solutions. Further functionalization of selected structured surfaces is realized through hydrophobization using chemical vapor deposition of a fluorinated silane agent. Pool boiling experiments are conducted in the saturated state and at atmospheric pressure. The contribution of each employed enhancement technique to boiling performance is analyzed and discussed to determine how various enhancement methods could be combined and what the risks are for deterioration of the overall performance due to unforeseen effects.

## 2. Materials and Methods

### 2.1. Surface Functionalization and Evaluation

The study was carried out on samples made of electrolytically pure copper (ECu, > 99.9% Cu). Each sample was manufactured from a 25 mm rod, and the geometry of the sample is shown in the Supplementary Materials Figure S1b. The top flat face of the sample, machined to a diameter of 14 mm, served as the active boiling surface and was optionally functionalized. All surfaces were first sanded with P1200 and then P2000 grit sandpapers to reduce the surface roughness to approx. *S*_a_ = 0.15 µm. The surface was then rinsed with 2-propanol and wiped several times with lint-free wipes to remove any contaminants. All samples received this pretreatment, and the reference sample (“REF”) was tested in the described condition without undergoing any additional surface treatment.

#### 2.1.1. Type A Surfaces

Two types of surfaces with significantly different surface morphology and chemical composition were prepared with the aim of boiling heat transfer enhancement. A chemical oxidation procedure adopted from other works [18,20,21,22,27,36] was employed to prepare surfaces denoted with the letter “A”. First, the sample was submerged face-down into a 5 M HCl bath for 2 min to remove the native oxide layer from the surface. Afterward, it was immersed into a beaker of fresh water for at least 3 min to stop all reactions. Finally, the sample was submerged face-down into an alkaline solution for 10 min. The solution was composed of NaClO_2_, NaOH, Na_3_PO_4_ 12H_2_O and H_2_O in a ratio of 3.75:5:10:100 by weight, respectively. The temperature of the solution was kept at 95 ± 3 °C for the duration of the treatment using a hotplate. The sample was removed from the beaker after the treatment was complete, rinsed with distilled water and again submerged in distilled water for 5 min before being dried in a convection oven for 15 min at approx. 80 °C.

#### 2.1.2. Type B SurfacesFigure

Surfaces denoted with the letter “B” were prepared via a modified method of controlled chemical oxidation, as described in various sources [18,23,24,25,26,73]. The method was adopted to create oxidized microstructured surfaces potentially suitable for boiling performance enhancement. The surface functionalization procedure was exactly the same as for type A surfaces; however, 1 M NH_3_ H_2_O (alternatively NH_4_OH) was used as an alkaline solution, and the oxidation was conducted at 60 °C for 2 h.

#### 2.1.3. Hydrophobization

Selected surfaces were hydrophobized after the initial oxidation and subsequent drying. They are denoted with the addition of the letter “H”. The hydrophobization step was performed using chemical vapor deposition (CVD) of a fluorinated silane. Specifically, 0.05 mL of (heptadecafluoro-1,1,2,2-tetrahydrodecyl)trimethoxysilane and 0.01 mL of 1,2-bis(trimethoxysilyl)ethane were mixed with 0.95 mL of toluene in a small open vial, which was then placed in a 1 L container together with the sample to be hydrophobized. The container was sealed and kept in an oven at 90 °C for 90 min for the CVD process to occur. Afterward, the container was left to cool down before the samples were removed from it. No further treatment of the samples was performed following hydrophobization. The thickness of the fabricated monolayer coating was in the nanometer range [27].

#### 2.1.4. Naming Convention

Functionalized surfaces were denoted with letters based on the combination of the oxidation method (A or B) and hydrophobization (suffix H for hydrophobized surface and no suffix for non-hydrophobized surfaces). Additionally, some samples were produced in multiple copies to evaluate the repeatability of the fabrication process and for selected experiments with different fluids.

#### 2.1.5. Evaluation of Surface Properties

Surface morphology was analyzed using scanning electron microscopy (SEM). The analysis was performed using Carl Zeiss SUPRA 35 VP SEM, operating at 1 kV accelerating voltage and utilizing the secondary electron detector. SEM imaging was performed on a set of identical surfaces to avoid damaging the surfaces intended for boiling experiments. In selected cases, additional SEM imaging was performed after the boiling experiments to observe surface morphology changes.

The changes in surface chemistry after certain experiments with self-rewetting mixtures of 1-butanol and water were investigated using Raman spectroscopy to determine surface composition and possible contamination. WiTec Alpha 300R Raman microscope with a 532 nm laser was used for this purpose. The measurements were performed at low and medium laser power using a 100x objective. The diameter of the sampled spots was approx. 250–500 nm and the depth of the analysis was between 2–5 µm. The spectra were acquired in the wavenumber range between 70 and 3600 cm^−1^, although the higher end of the spectrum offered poor signal-to-noise ratio. The acquired spectra were used to identify copper species on the surface and detect the presence of any contaminants. Most authors mention the position of Cu_2_O peaks to be at approx. 220 cm^−1^ (main peak), 520 cm^−1^, 570 cm^−1^, 620 cm^−1^ and 790 cm^−1^, with the peaks between 500 and 630 cm^−1^ forming a “hump” [74,75,76,77,78,79]. On the other hand, CuO peaks mostly appear at 298 cm^−1^ (main peak), 340 cm^−1^ and 630 cm^−1^ [76,77,79,80]. Finally, Cu(OH)_2_ exhibits a peak at approx. 300 cm^−1^ and a main peak at 490 cm^−1^ [79,81,82,83,84]. These values were used to identify possible changes in the chemical composition of the surface due to transition between copper species. Since the exposure of copper (and similar materials capable of acting as catalysts) to n-butanol at elevated temperatures, which can appear during boiling, may cause oxidation and conversion to other hydrocarbon species (such as n-butyraldehyde) [85,86,87,88], the spectra of possible reaction products were also reviewed [89,90,91,92] to determine whether such species might have reacted with or formed on the surface.

Surface properties were also evaluated through wettability measurements. Specifically, the static contact angle was measured on each type of surface before its exposure to boiling. For this purpose, a custom goniometer setup was used, and the measurements were conducted using distilled water or Novec 649™ at room temperature. Contact angle measurements were not performed for water/butanol mixtures due to preferential evaporation of butanol, which was reported to impact the measured values [93]. At least five droplets with a volume of 15–20 µL were deposited on different parts of the surface and their images were recorded using a camera. The images were then processed using a custom-developed algorithm to determine the values of static constant angles.

### 2.2. Boiling Performance Evaluation

Pool boiling performance of developed surfaces was evaluated using a previously developed experimental setup and measurement protocol [94]. The setup is shown in the Supplementary Materials Figure S1. Data reduction was performed in accordance with suggestions made in [95]. Briefly, the functionalized surfaces were evaluated under pool boiling conditions at atmospheric pressure. Measurements of temperatures within the sample allowed for the calculation of heat flux, superheat and HTC. Details on the design of the experimental setup can be found in Section S1 of the Supplementary Materials. Data reduction methods are presented in Supplementary Materials Section S2 together with evaluation of measurement uncertainty. Finally, measurement protocols are described in Supplementary Materials Section S3.

## 3. Results and Discussion

### 3.1. Functionalized Surfaces

**Figure 2:**
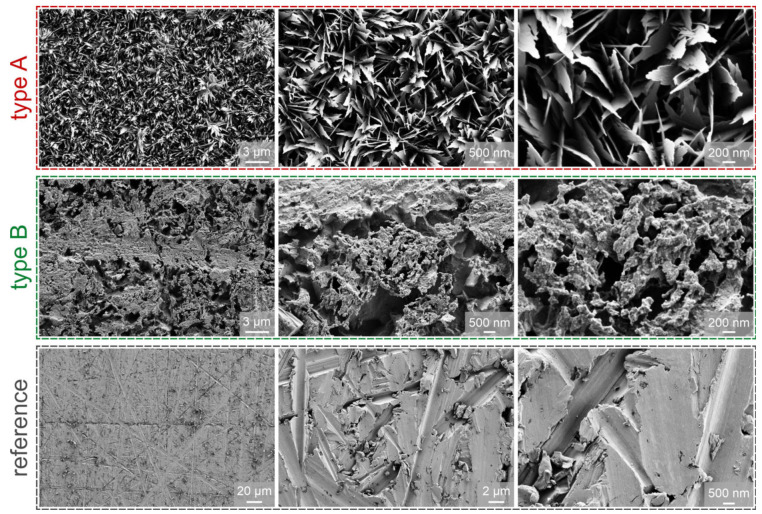
SEM images of type A surface (**top**), type B surface (**middle**) and the reference surface (**bottom**).

In this section, the properties of functionalized surfaces are discussed and compared with existing studies. SEM images of the two types of functionalized surfaces are shown in Figure 2 alongside the images of the untreated reference surface.

#### 3.1.1. Type A Surfaces

**Figure 3:**
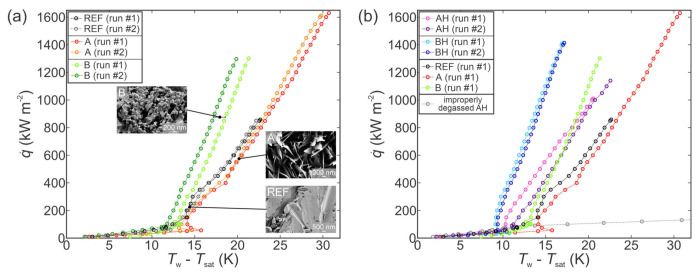
Boiling curves using water on the chemically oxidized surfaces (**a**) and comparison with their hydrophobized variants (**b**).

During this treatment, a thin layer of Cu_2_O is formed via oxidation, which quickly reoxidizes to CuO. Reoxidation, i.e., the growth of hydrophilic CuO nanostructures on the Cu_2_O layer, starts after approximately 1 min, and a dense array of CuO with height of approx. 1 µm is obtained after only 5 min [22,73]. As the oxidation continues, the thickness of passivating CuO layer increases and the oxide growth rate decreases, which makes the process self-limiting and useable for uniform nanocoating of various complex microstructures [20,22,36]. After the procedure, sharp, knife-like structures of CuO are obtained on the surface, and the sample turns completely black [73]. The layer is approximately 1 µm thick and has a solid fraction of about 0.04 [27]. Underneath the layer, there is a thin Cu_2_O layer with thickness of approximately 300 nm [18,22,27]. Thermal resistances of both layers were reported to be negligible [20,21]. Type A surface structures are characterized with a high roughness factor and high capillary performance due to their sponge-like morphology [37,73]. In this study, long treatment time of 10 min was applied to ensure a complete and uniform formation of surface nanostructures presented in Figure 3.

#### 3.1.2. Type B Surfaces

During the controlled oxidation treatment of type B surfaces, Cu(OH)_2_ is formed and then converted into CuO. Temperatures of at least 60 °C are required for the conversion to occur [16]. A hierarchical structure characterized by high roughness factor and sponge-like morphology of nest-like microspheres with diameters of several µm constructed of branched uniform smooth and thin nanowalls with thickness in the order of 10 nm is supposed to be created by this procedure [18,25,73]. The process should not be self-limiting, as longer oxidation time leads to more thick and complex oxide structures [18]. The resulting hierarchical structure was reported not to be suitable for boiling applications, as lower HTC and decreased number of nucleation sites were reported with respect to bare copper despite the increase in roughness by an order of magnitude due to the presence of microballs [23]. It was reported that when nanoribbons of Cu(OH)_2_, which are crucial for the resulting CuO structure [25], are kept in the oxidation solution for longer, they become easy to peel off and deposit on the bottom of the oxidizing bath [26,94]. When this happens, there is no film on the surface, and the copper is continuously oxidized and dissolves into the alkaline solution which then becomes blue in color [94]. Furthermore, it was reported that the structure of Cu(OH)_2_ is significantly affected by various factors of pretreatment, such as the coarseness of the grinding paper, direction of grinding, time spent in HCl bath and others [95]. The length of Cu(OH)_2_ structures also varies with reaction temperature and time [96].

A structure different from the microball/nanowall structure was obtained after chemical oxidation in the present work. SEM images in Figure 3 show that the structure of type B oxidized surfaces resembles surface structures produced after chemical etching and that microcavities suitable for boiling are present on the surface. Microcavities obtained on type B surfaces have diameters in the range from 40 nm to 2 µm, but even cavities with a diameter above 10 microns can be seen in Figure 2. Such cavities and other irregularities on the micron and submicron scale were reported to have a positive effect on boiling performance, especially on HTC and ONB [15,29,31,97,98]. Neither microballs nor any visible nanostructure were present on the surfaces after chemical oxidation via the type B process. This could be due to causes mentioned above. The blue color of the solution was observed during type B oxidation. It is therefore possible that Cu(OH)_2_ structures fell off the surface before their conversion to CuO due to different pretreatment, face-down orientation of the surface, time of oxidation or other factors.

#### 3.1.3. Surface Wettability

The wettability of all fabricated surfaces was evaluated through static contact angles with sessile drop measurements. The measured contact angle values averaged for multiple copies of the same surface for water and Novec™ 649 are listed in Table 1 alongside their standard deviation. While significant differences in contact angle values of different surfaces were obtained for water, the contact angles recorded with Novec™ 649 were very similar for all investigated surfaces.

In literature reports, the contact angle on surfaces similar to type A surface was observed to continuously decrease with increasing time of oxidation. A decrease from the initial value of about 90° to 25° and to 6° was reported after 3 and 5 min of oxidation, respectively [18]. The wickability of the resulting CuO nanostructure depends on the microstructure of the surface. CuO nanostructured surfaces are typically strongly hydrophilic or superhydrophilic [18,20,21,36]. Overall, type A surface exhibited the lowest contact angles and the highest affinity to the liquid droplets, both with water and Novec™ 649. This can be ascribed to the CuO nanostructures on the surface, which greatly increase the submicron surface roughness and facilitate liquid spreading. After hydrophobization of type A surface, which is denoted as “AH”, superhydrophobic behavior was observed with contact angles above 150° and a roll-off angle below 5°. However, for Novec™ 649, which has a much lower surface tension than water (approx. 10.8 mN m^−1^), only slightly increased contact angles were obtained on hydrophobized surfaces.

Type B surfaces exhibited slight hydrophobicity after the chemical oxidation treatment. In the hydrophobized state, the contact angle of water increased to 152°, but the surfaces did not exhibit a roll-off angle, meaning they cannot be classified as superhydrophobic. The contact angles of Novec™ 649 on the B and BH surfaces are comparable to those on the A and AH surfaces; the application of the low-surface-energy coating increased the contact angle by about 2°.

Compared with the functionalized surfaces, the untreated reference surface (“REF”) exhibited a neutral contact angle with water (91°), while the Novec™ 649 contact angle also fell in between those recorded for the otherwise-hydrophilic surface A and its hydrophobized variant AH.

### 3.2. Boiling Heat Transfer with Water

#### 3.2.1. Comparison of Boiling Curves and Heat Transfer Coefficients

The boiling performance of type A and type B surfaces was first evaluated using pure water in the “as prepared” state with no hydrophobic coating. Figure 3a shows a comparison of their boiling performance with the untreated reference surface. For all three surfaces, the first and second measurements of the boiling curve are shown to account for the potential scatter.

Both chemically oxidized surfaces exhibited enhanced heat transfer in comparison with the untreated surface. Specifically, the highest CHF of 1630 kW m^−2^ (enhancement of 90%) was recorded on type A surface, which also exhibited the lowest contact angle. Hydrophilic behavior typically results in increased CHF values due to enhanced rewetting of the surface following bubble departure and prevents excessive vapor spreading and consequent local dryout [99,100,101]. However, higher superheat is generally necessary for nucleation on hydrophilic surfaces, resulting in lower HTC values, which was also observed during our experiments and is shown in Figure 3a. Type B surface also enhanced the CHF (by 52%), which can be attributed to abundant microcavities that formed on the surface. These serve as active nucleation sites from which vapor bubbles form and grow [102]. Their diameters were confirmed to fall into the optimal size range for nucleation-promoting (micro)cavities, as predicted by Hsu’s nucleation criterion [97] or Kandlikar’s criterion [103]. These criteria predict that in the case of saturated pool boiling of water at atmospheric pressure, the optimal range of microcavity radii for efficient nucleation is approx. 0.1–100 µm. Furthermore, since the presence of microcavities suitable for nucleation increases the nucleation site density, the HTC is also enhanced.

The obtained results agree with observations previously made by other authors. Raman and McCarthy [36] used the same type A treatment process and obtained enhanced CHF of 1.96 MW m^−2^ compared with a CHF of 1.17 MW m^−2^ measured for pure copper. They explained this as being caused by the increased wickability of type A surface. However, the superheat at CHF of 34.5 K was significantly higher compared with 16 K obtained for their reference surface, and maximum HTC of 57 kW m^−2^ K^−1^ was lower for type A surface than 70 kW m^−2^ K^−1^ measured for the reference surface. This agrees with our recorded values of surface superheat near the CHF, as we also observed values in excess of 30 K. Raman and McCarty ascribed the decrease in HTC over the entire boiling curve to the suppression of active nucleation sites due to the presence of nanostructures on the surface, as they reduced the cavity size below 1 µm. Chu et al. [21] also used the type A oxidation process for surface functionalization and obtained a CHF enhancement of up to 200% for hierarchical surface with type A nanostructure. The performance increase was attributed to increased roughness, which pins the contact line of emerging bubbles. Similarly, microcavity surfaces were previously shown to considerably enhance the boiling performance, especially the HTC [41,104,105]. Using patterned microcavity surfaces, Voglar et al. [106] achieved an HTC of 54.6 kW m^−2^ K^−1^ on laser-textured stainless steel. Similarly, Može et al. [107] produced microcavities in the size range of approx. 0.4–5 µm using laser texturing on copper surfaces and increased the HTC from 33.3 kW m^−2^ K^−1^, as recorded on an untreated surface, to 76.1 kW m^−2^ K^−1^, as recorded on the best-performing functionalized surface.

The results of boiling performance evaluation of hydrophobized surfaces (AH and BH) are shown in Figure 3b and compared with results obtained on the chemically oxidized surfaces and the reference surface. Firstly, it is evident that improper degassing of a hydrophobized surface will result in a false early transition toward film boiling due to the entrapped air on the surface, as reported by Allred et al. [42]. However, when properly degassed, both hydrophobized surfaces outperformed their chemically oxidized counterparts in terms of an early onset of nucleate boiling and higher HTC values. Nevertheless, lower surface energy due to the fluorinated silane coating also results in less intense rewetting of the surface due to its higher affinity for contact with the vapor phase, which in turn results in decreased CHF values. This is evident from decreased CHF values recorded on the AH surface in comparison with the non-hydrophobized type A surface. However, this did not affect the CHF behavior of the hydrophobized surface BH where a slight increase in the CHF was observed and can be attributed to the aforementioned abundance of active nucleation sites guaranteed by the microcavities and a consequent absence of major vapor spreading due to horizontal coalescence of the bubbles.

**Figure 4:**
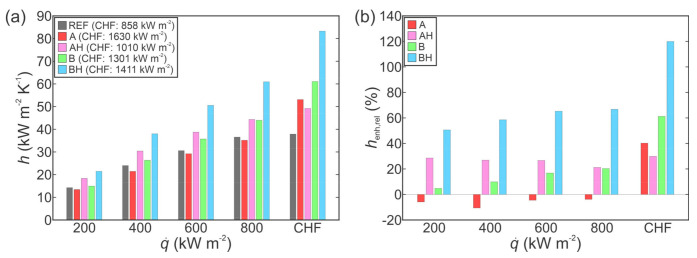
Comparison of HTCs for the boiling of water in absolute terms (**a**) and relative to the reference surface (**b**).

The increased heat transfer performance of functionalized surfaces is further evident from the comparison of HTC values at selected heat fluxes, as shown in Figure 4a in absolute terms and in Figure 4b relative to the performance of the untreated reference surface. In all cases, the results of the first run on a given surface are compared. Firstly, the non-hydrophobized type A surface actually exhibited lower HTC values compared with those recorded on the reference surface at the same heat flux. Only at very high heat fluxes, which were not achieved on the reference surface due to earlier transition to film boiling, did type A surface provide superior HTC values. Non-hydrophobized type B surface mostly exhibited a modest enhancement of heat transfer performance below 20%. However, near CHF, significantly higher HTC of 61 kW m^−2^ K^−1^ was recorded, which is a major enhancement of 61% over the reference surface. At all heat fluxes, both hydrophobized surfaces (i.e., AH and BH) exhibited a major enhancement of the HTC. The highest HTC value was recorded on the BH surface at the point of CHF incipience, with HTC reaching 83 kW m^−2^ K^−1^, which represents a 120% enhancement over the highest HTC recorded on the reference surface.

The boiling performance enhancement and associated mechanisms can be explained in several steps. Firstly, the temperature of the onset of nucleate boiling (ONB) should be primarily minimized to increase the HTC at the early stage of the nucleate boiling regime [108]. Secondly, the nucleation frequency, nucleation site density and liquid suction toward the nucleation sites should also be increased as higher heat fluxes (i.e., when nucleate boiling is well developed), in order to fully utilize the entire boiling surface and maximize vapor production at a given heat input. Finally, the onset of CHF conditions should be delayed through the prevention of horizontal bubble coalescence that results in the formation of large dry-out areas [14,109,110]. The minimization of ONB temperature and optimization of cavity sizes, which act as active nucleation sites, can be explained through Hsu’s nucleation criterion [97]. In theory, the existing vapor embryo will start to grow from a certain surface cavity at any given wall temperature (or heat flux) if the cavities’ diameter falls within a certain range.

The results indicate that functionalized surface B generally provides higher HTC and lower ONB compared with type A surface and the untreated reference surface, which can be attributed to the abundance of microcavities on the surface, as observable in Figure 2. In this context, the cavities of type B surface cover the entire range of suitable cavity sizes, as predicted by Hsu’s criterion for given operating conditions. On the other hand, type A surface includes randomly distributed and vertically grown CuO knife-like nanostructures. In this case, surface roughness is significantly increased, but less prone to form vapor-trapping cavities that are observed on type B surface. Consequently, type A surface offers a slight HTC enhancement compared with the reference sample, but is not superior over the type B sample, mainly because of less potentially active nucleation sites.

Another important parameter in boiling performance is surface wettability. Hydrophobization of type A and B samples significantly decreased surface wettability and rendered both samples superhydrophobic. This effect shifted the range of optimal cavity diameters to higher values, according to Hsu’s nucleation criterion, but also increased the ability of surfaces to entrap gas (air or vapor). Higher amount of vapor embryos further increased the HTC in the entire range of nucleate boiling regime for AH and BH samples, as is clearly demonstrated in Figure 3b.

Finally, the porosity and the wicking ability are also an important surface parameter that affect boiling performance. It is generally accepted that wicking surface structures (i.e., surfaces with certain porosity and intrinsic hygrophilicity [111]) enhance CHF for different fluids [14,112]. This is due to the rapid supply of fresh liquid toward active nucleation sites through the wicking structures and higher resistance against the entire microlayer evaporation underneath growing bubbles. The movement of liquid in the porous media can be explained, for example, by the existing hemispreading model [113]. Experimental results showed that the speed of water through the nanoporous (wickable) media was in the order of 10–100 mm s^−1^. Our results in Figure 3b show that highest CHF was achieved with type A surface due to its intrinsic hydrophilicity and significantly higher porosity compared with samples B and REF. The wicking ability of type A surface is reduced after hydrophobization (sample AH), and the CHF is also reduced.

The results recorded on both the untreated and selected functionalized surfaces were compared with well-known correlations for predicting CHF and HTC values. The experimental results obtained on untreated surfaces match the theoretical predictions well. A complete evaluation is provided in the Supplementary Materials in Section S4 (Table S1 and Figure S2).

To summarize, all four functionalized surfaces exhibited enhanced boiling heat transfer with water through increased HTC and/or CHF values. Due to the most promising characteristics of the non-hydrophobized type A surface (on which the highest CHF was recorded) and the hydrophobized surface BH (on which the highest overall HTC was recorded), most attention was given to these two types of surfaces in the following experiments.

#### 3.2.2. Repeatability of Surface Functionalization and Surface Aging

To evaluate the repeatability of the surface functionalization process, multiple copies of selected surfaces were prepared, and their boiling performance was evaluated under the same conditions. In addition to the reference surface, the repeatability tests were performed on surfaces with most favorable heat transfer characteristics, which were identified in Section 3.2.1 (i.e., surfaces A, B and BH). The results of this evaluation are presented in the Supplementary Materials in Section S5 and in Figure S3. Overall, the fabrication repeatability is deemed as satisfactory, since multiple copies of different boiling surfaces exhibited comparable boiling performance.

In realistic applications, boiling surfaces would be exposed to the boiling process for prolonged periods of time, yet their aging performance is often neglected [19]. Therefore, the selected surfaces (the same as in the surface functionalization repeatability analysis) were exposed to multiple subsequent boiling curve measurements, with each one carried out until CHF incipience and subsequent transition toward film boiling. The results of surface aging evaluation are presented in the Supplementary Materials in Section S6 and compared in Figure S4. Overall, while some aging effects were observed on non-hydrophobized surfaces, all functionalized surfaces are deemed suitable for long-term exposure to boiling water.

### 3.3. Boiling Heat Transfer with Self-Rewetting Fluids

Following the evaluation of boiling performance of developed surfaces with pure water, another set of experiments was performed to assess (i) how a working fluid with self-rewetting properties influences boiling heat transfer on functionalized surfaces and (ii) whether a self-rewetting fluid (SRF) could be used to further enhance boiling performance already improved through surface engineering. In all cases, experiments with mixtures of 1-butanol (BuOH) and water were performed for the range of mass concentration of BuOH from 1 to 6 wt.%. First, stable performance was achieved with pure water and then experiments with BuOH mixtures were started by increasing the concentration of 1-butanol in steps of 1 or 2 wt.%. After achieving 6 wt.% of BuOH, the measurements were repeated for 0, 2, 4 and 6 wt.% to verify whether the boiling surface and its performance remained stable. Details on the exact methodology are provided in the Supplementary Materials in Section S3.

#### 3.3.1. Effect of Butanol Concentration on Boiling Performance and Boiling Curve Shift

**Figure 5:**
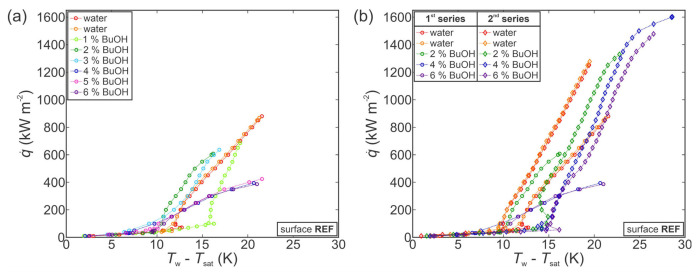
Boiling performance of the reference surface (REF) during the first series of measurements with a self-rewetting fluid (**a**) and comparison of selected concentrations from the first and the second series of measurements (**b**).

First, the experiments were performed on an untreated surface. The results for the first series of measurements are shown in Figure 5a. As evident from the two boiling curves for pure water, the boiling performance was in a stable state prior to measurements with SRFs. Adding 1 wt.% of BuOH shifted the boiling curve toward slightly higher superheats and reduced the CHF to approx. 700 kW m^−2^. Further additions of BuOH then caused the boiling curve to shift toward lower superheats in comparison with water and 1 wt.% measurements, while also further reducing the CHF value. Finally, at BuOH concentrations of 4 wt.% or more, the boiling curves became flattened and a heat flux of only about 400 kW m^−2^ was achieved before a gradual transition into film boiling. This inconsistent behavior might indicate that the surface underwent a chemical reaction with the 1-butanol present in the working fluid, which significantly changed the surface properties and boiling behavior. This is discussed further in Section 3.3.2.

Similar boiling performance was obtained through a second series of measurements on the same surface, which are shown in Figure 5b. It is evident that the boiling performance with water improved significantly after the surface was exposed to the first series of measurements with water/butanol mixtures. Furthermore, measurements with 2, 4 and 6 wt.% of BuOH within the second series of experimental runs do not correspond at all with those recorded during the first series, with both the boiling curves and general trends being completely different. This confirms the initial observation that the untreated surface appeared to be unstable in combination with SRFs, and it underwent chemical reactions that fundamentally changed its boiling behavior. Some studies suggest that copper or copper-containing compounds can successfully be used as catalysts for dehydrogenation of 1-butanol to butyraldehyde (i.e., butanal) [85,87,88]. This happens at elevated temperatures (typically above 200 °C), which were reached during our experiments after CHF incipience and transition toward film boiling. It is therefore likely that after several CHF onsets and several subsequent periods of exposure to high temperature, the copper boiling surface reacted with 1-butanol to form butyraldehyde or similar species.

These results indicate that copper surfaces might not be suitable as boiling interfaces when SRFs are used due to the ensuing chemical reactions. While many studies conducted with untreated or functionalized copper surfaces in combination with SRFs can be found in the literature, they did not address possible significant changes in surface properties, structure and chemistry, to the best of our knowledge.

**Figure 6:**
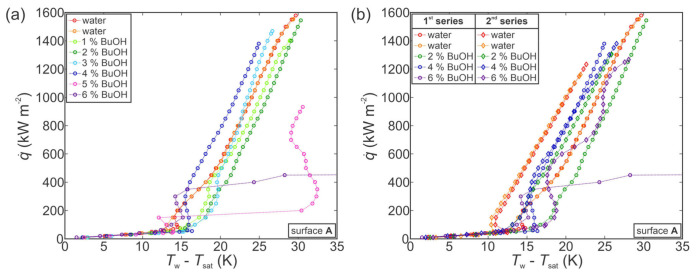
Boiling performance of type A surface during the first series of measurements with a self-rewetting fluid (**a**) and comparison of selected concentrations from the first and the second series of measurements (**b**).

With the previously observed change of boiling performance in mind, further tests were conducted with functionalized surfaces to evaluate their degradation during exposure to SRFs. Figure 6 shows the results obtained during measurements on a type A surface. During the first series of measurements (see Figure 6a), the boiling curve first shifted toward higher superheats with a small decrease in CHF at a concentration of 1–2 wt.% of BuOH. When the concentration was increased, a similar shift was observed as in the case of the untreated reference surface (compared with Figure 5a), with decreased superheats and decreased CHF values. This was followed by significant degradation of boiling performance at a concentration of 5 and 6 wt.% of BuOH, where CHF was first reduced below 1000 kW m^−2^ (at 5 wt.%) and then to below 500 kW m^−2^ at 6 wt.%. This, again, indicates that the boiling surface might have undergone a chemical reaction with the butanol in the SRF. Reaction with water is unlikely, since the surface previously exhibited excellent stability during 13 consecutive experimental runs with water, as was shown in the Supplementary Materials Figure S4b.

A second series of measurements confirmed our previous observations. A comparison of the first and second series of measurements for pure water and 2, 4 and 6 wt.% of BuOH mixtures is shown in Figure 6b. The boiling performance with pure water noticeably changed after exposure to SRFs during the first series of measurements with CHFs and superheats reduced by approximately 25% and 5 K, respectively. Repeated measurements at 2, 4 and 6 wt.% of BuOH followed the same trend as for the second series of measurements on the untreated reference surface, with increased CHF values and increased surface superheats compared with the measurement with water at the beginning of the second series.

Finally, the use of SRFs was evaluated on the hydrophobized surface BH, which previously exhibited the highest HTC values. The focus was placed on whether the hydrophobic coating can protect the surface from degradation observed on the untreated and type A surfaces, and whether boiling performance can be enhanced both in terms of higher CHF and concomitantly higher HTC values.

**Figure 7:**
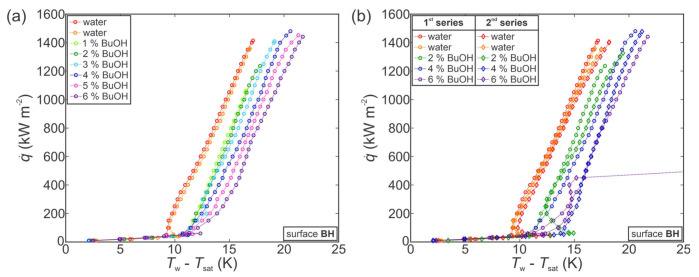
Boiling performance of hydrophobized surface BH during the first series of measurements with a self-rewetting fluid (**a**) and comparison of selected concentrations from the first and the second series of measurements (**b**).

Figure 7a shows the first series of measurements performed with pure water and then with 1–6 wt.% of BuOH. At low concentrations of 1–2 wt.% of BuOH, the same shift of the boiling curve is observed as with previously shown measurements on the untreated reference surface of type A surface. The boiling curve is slightly shifted toward higher superheats and the CHF is decreased. At higher concentrations, however, different trends are observed for the BH surface. At 3 wt.% or more, the CHF increases back to the original value for water, while the boiling curve is still shifted toward higher superheat by about 0.5 K per added wt.% of BuOH. An increase in ONB temperature is also observable with approx. 2 K higher surface superheat required for nucleation to start when SRFs are used instead of water.

The second series of measurements, shown in Figure 7b, matches the results from the first series, confirming that little-to-no degradation of the boiling surface had taken place. Similar behavior in terms of CHF values decrease and superheat increase is also observed for 2 and 4 wt.% measurements. However, the second series of measurements at 6 wt.% exhibited severe degradation of boiling heat transfer with a significant drop in CHF to below 500 kW m^−2^. This matches the behavior of the reference and type A surfaces during the first series of measurements with water/butanol mixture, indicating chemical changes of the surface after a certain number of runs, which might have been caused by continuous degradation of the hydrophobic coating.

Additional measurements performed on another copy of the hydrophobized surface BH (see Supplementary Materials Section S7 and Figure S5) confirmed that the fluorinated silane coating at least temporarily protects the surface against reaction with 1-butanol.

#### 3.3.2. Surface Chemistry and Morphology Changes

SEM imaging and Raman spectroscopy were performed to investigate the significant changes in boiling performance after exposure of certain surfaces to the boiling of water/butanol mixtures. Non-hydrophobized type A surface was selected for this purpose, as the change in boiling performance was the most pronounced on it, and since the surface chemical composition resulting from the chemical oxidation treatment was known in advance.

**Figure 8:**
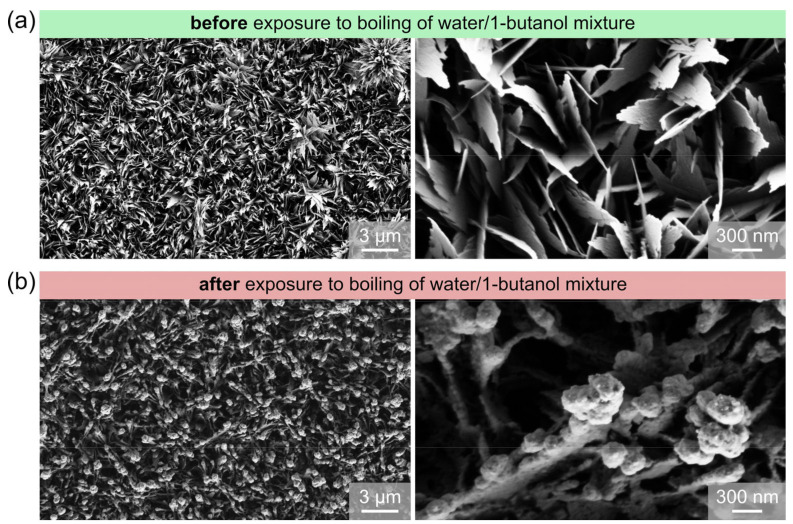
SEM images of type A prior to experiments (**a**) and after exposure to the boiling of water/1-butanol mixture (**b**).

SEM images of type A surface before and after the exposure to the boiling of water/butanol mixture are compared at same magnifications in Figure 8. It is evident that the CuO knife-like nanostructure, which forms during the type A oxidation process in the alkaline bath, is significantly transformed after the boiling experiments with BuOH mixtures. Specifically, the thin and sharp structures are transformed into rounded structures with a pronounced spherical end. This confirms that the CuO oxide on the surface underwent a chemical reaction with 1-butanol.

**Figure 9:**
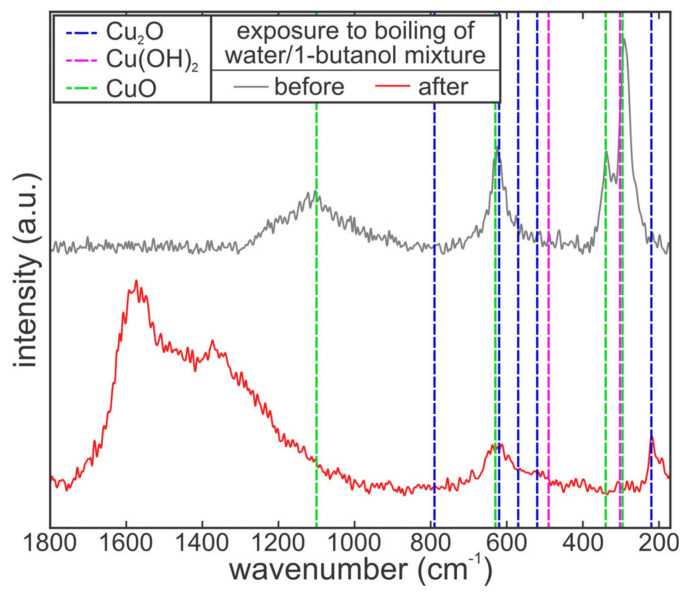
Raman spectra recorded on type A surface before and after the boiling of water/1-butanol mixture. Main peaks of typical copper compounds correspond to wavenumbers of 220 cm^−1^ for Cu_2_O, 298 cm^−1^ for CuO and 490 cm^−1^ for Cu(OH)_2_.

To better evaluate what chemical species formed as a result of exposure of the CuO nanostructures to BuOH mixtures, Raman spectroscopy of type A surface was conducted on type A surface after exposure to the boiling of BuOH/water mixtures and on an additional sample not exposed to boiling. Even though Raman spectroscopy is a relatively deep method of surface chemistry analysis, with a sampling depth of several micrometers, the results provide reliable data regarding the oxide, hydroxide and similar compounds in the uppermost layers of the surface (i.e., the top micrometer or so). The comparison of Raman spectra in the 180–1800 cm^−1^ wavenumber range is shown in Figure 9. Additionally, the positions of typical peaks for CuO, Cu_2_O and Cu(OH)_2_, as identified based on the literature, are shown to allow for easy identification of copper compounds. The Raman spectra of the sample not exposed to boiling (grey line) clearly show the presence of CuO on the surface, forming peaks at approx. 298 cm^−1^ (main peak), 340 cm^−1^, 630 cm^−1^ and 1100 cm^−1^. After exposure to the boiling of water/butanol mixtures, however, these peaks disappear from the spectrum (red line). Instead, two smaller peaks, indicating the presence of Cu_2_O, appear at approx. 220 cm^−1^ and 620 cm^−1^, indicating the deoxidation of CuO and transformation into Cu_2_O. Additionally, a major broad hump appears in the wavenumber region between 120–1700 cm^−1^, with two observable peaks at approx. 1370 cm^−1^ and 1570 cm^−1^.

When comparing these data to typical spectra of 1-butanol and compounds formed from it through simple reactions (e.g., butyraldehyde and butyric acid), no good match could be established. Nevertheless, this hump in the spectrum is certainly caused by carbon-containing compounds on the surface. The closest matches might be butyraldehyde, with its peak at approx. 1396 cm^−1^ (typically produced through dehydrogenation of 1-butanol), or 1-butanol itself with peaks at approx. 1300 cm^−1^ and 1452 cm^−1^. In any case, the results further confirm that a chemical reaction of the surface with 1-butanol took place, which changed both the surface chemistry and morphology and in turn affected the heat transfer characteristics.

### 3.4. Boiling Heat Transfer with Novec™ 649

Following the evaluation of boiling performance on developed functionalized surfaces using water and water/butanol mixtures, another set of measurements was conducted on fresh copies of each surface to evaluate their heat transfer performance with a novel dielectric fluid Novec™ 649. Since the properties (especially the surface tension) of this dielectric fluid differ significantly compared with water, different trends to those identified for water and water-based SRF mixtures were expected.

**Figure 10:**
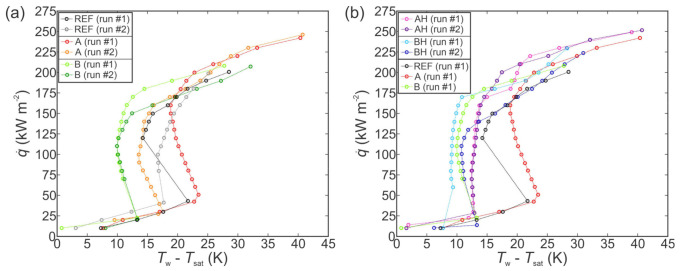
Boiling curves recorded for Novec™ 649 on the fabricated surfaces (**a**) and comparison with their hydrophobized variants (**b**).

First, the boiling performance of the chemically oxidized type A and B surfaces was evaluated and compared with the performance of an untreated surface. These results are shown in Figure 10a, from which it is evident that differences between the boiling curves for different surfaces are much smaller than those observed during experiments with water. However, a similar trend can be identified with type A surface exhibiting the highest CHF value, which can be ascribed to the lowest contact angles (although according to Table 1, the differences in the wettability of functionalized surface with Novec™ 649 are very subtle) and the surface nanostructure enabling enhanced rewetting. Similar to water, type B surface enhanced the HTC and caused the onset of nucleate boiling at a noticeably lower surface superheat with a minor increase in the CHF value. Somewhat larger deviations between the first and the second boiling curve recorded on an individual surface were observed for the boiling of Novec™ 649, which was further investigated during the aging evaluation (Supplementary Materials Section S8).

Hydrophobized variants of both functionalized surfaces were also tested with Novec™ 649. While neither of these surfaces exhibited “phobicity” toward Novec™ 649 due to its low surface tension, the measured contact angles (Table 1) indicate that the application of the fluorinated silane coating slightly increased the contact angle. Therefore, an impact on the boiling performance is also expected and was indeed observed in the results of experimental runs shown in Figure 10b. Hydrophobized surface BH exhibited the lowest ONB, and the boiling curve of the first experimental run also exhibited the lowest overall superheats. Similarly, hydrophobized surface AH exhibited significantly lower superheat values within the nucleate boiling regime (5–10 K compared with the non-hydrophobized type A surface). Interestingly, the CHF was not impacted in either case, with comparable CHF values observed for both pairs of hydrophobized and non-hydrophobized surfaces with the same previous oxidation treatment. This indicates that while the coating successfully promotes nucleation at lower surface superheats, importantly, the CHF incipience mechanism does not rely on the free surface energy.

**Figure 11:**
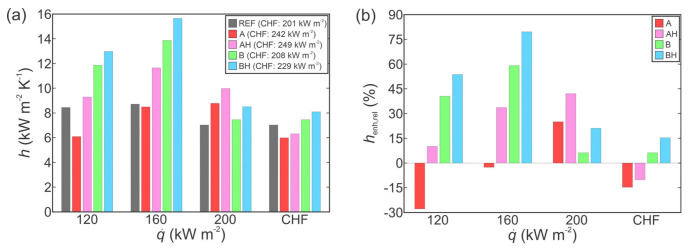
Comparison of HTCs for the boiling of Novec™ 649 in absolute terms (**a**) and relative to the reference surface (**b**).

Figure 11a compares HTCs measured on individual surfaces during the first experimental run at selected heat flux levels in absolute terms. HTCs with respect to the untreated surface are shown in Figure 11b. Contrary to the results obtained for water shown in Figure 4, the HTC is enhanced the most at low heat fluxes, whereas the enhancements of its value are smaller or non-existent close to the CHF. The latter is mainly the consequence of the boiling curve’s shape, since a “flat” top resembling a gradual transition toward the CHF (i.e., large superheat increases with small heat flux increases) is typical for the boiling of dielectrics. The highest HTC within this comparison was recorded on the hydrophobized surface BH (13.0 kW m^−2^ K^−1^ at 120 kW m^−2^), representing an enhancement of 54% compared with the untreated surface. The results also indicate that virtually no enhancement of the HTC was achieved at high heat fluxes (close to the CHF), with degradation observed in some cases. This, together with rather comparable CHF values, again, indicates that the surface structure might be less important in delaying the CHF onset when a low-surface-tension fluid exhibiting comparable contact angles on all surfaces is utilized. The major enhancement of the HTC observed at low heat fluxes proves the importance of nucleation-promoting microstructure, such as the microcavities on type B surfaces.

Surface tension of the fluid is important in influencing the size range of effective cavity diameter for efficient nucleation [97]. Lower surface tension of the fluid significantly reduces both the upper and lower limit of the effective cavity diameter. The same is also observed when reducing the contact angle of the surface. Results in Table 1 prove that the layer of fluorinated silane only slightly affects the contact angle of Novec^TM^ 649. As a result, the boiling enhancement on AH and BH samples compared with their non-hydrophobized counterparts was much smaller when the dielectric was used as a working fluid instead of water. Contrary to water, Figure 10 shows that hydrophobization has no significant effect on the CHF for Novec^TM^ 649. Nanoscale silane coating does not change the intrinsic wettability between the surface and the tested dielectric. Therefore, the coating alone has no observable effect on ONB, HTC and CHF for that type of fluid.

The aging of surfaces when exposed to the boiling of Novec™ 649 was evaluated the same way as for water experiments. Here, a noticeable degradation of HTC was observed in all cases, except for the hydrophobized surface AH. Detailed evaluation can be found in the Supplementary Materials (see Section S8 and Figure S6).

## 4. Conclusions

This study focused on the development of functionalized copper surfaces for enhanced pool boiling heat transfer by combining chemical oxidation treatment and subsequent hydrophobization of the surface. Boiling tests were performed with water, self-rewetting water/1-butanol mixtures and dielectric fluid Novec™ 649 to evaluate the boiling performance of developed surfaces in combination with fluids with significantly different properties. The following conclusions were drawn from the obtained results:

(1) Chemical oxidation combined with chemical vapor deposition (CVD) hydrophobization represents a facile way of functionalizing copper surfaces and inducing properties favorable for phase-change heat transfer enhancement.
(2) Functionalized surfaces exhibited increased heat transfer coefficient (HTC) and critical heat flux (CHF) values in comparison with the untreated reference surface. HTCs of 83 kW m^−2^ K^−1^ and 13 kW m^−2^ K^−1^ were obtained using the hydrophobized surface BH with oxidation-induced microcavities with water and Novec™ 649, respectively. This represents and enhancement of 120% and 54% compared with the untreated surface operating at the same heat flux, respectively. The same functionalized surface increased the CHF value by 64% and 14% for water and Novec™ 649, respectively.
(3) Surface fabrication repeatability was found to be favorable with only minor differences in performance between copies of the same surface. Surfaces were also shown to be stable in combination with boiling water but exhibited significant negative aging due to exposure to the boiling of Novec™ 649 in most cases.
(4) Performance of self-rewetting fluids (SRFs) based on mixtures of water and 1-butanol at different concentrations boiling on functionalized copper surfaces was found to be unpredictable and problematic. Heat transfer performance was mostly degraded when SRFs were used instead of pure water. Furthermore, changes in surface morphology and in the chemical composition of the surface were detected.
(5) The (hydrophobic) fluorinated silane coating at least temporarily protected the oxidized copper surfaces from chemical reactions with water/1-butanol mixtures or Novec™ 649.

The results of the study indicate that more attention should be paid to degradation of surfaces and their boiling performance due to aging and/or contact with various fluids.

## Figures and Tables

**Table 1:** Contact angles on fabricated surfaces recorded with different fluids.

Surface	Water Contact Angle	Novec™ 649 Contact Angle
REF	91° ± 5°	20.0° ± 0.8°
A	14° ± 7°	17.1° ± 1.2°
AH	164° ± 2°	21.3° ± 1.7°
B	113° ± 7°	17.2° ± 1.6°
BH	152° ± 4°	19.2° ± 1.1°

## Data Availability

Data are available from the authors upon reasonable request.

## Supplementary Materials

The following are available online at https://www.mdpi.com/article/10.3390/nano11123216/s1, Section S1. Experimental Setup for Boiling Performance Evaluation [Figure S1. Experimental setup (a) and cross-section of the sample on heating assembly (b)]; Section S2. Data Reduction and Measurement Uncertainty; Section S3. Measurement Protocols for Boiling Performance Evaluation; Section S4. Comparison of Boiling Performance with Established Models [Table S1. Thermophysical properties of water and Novec™ 649; Figure S2. Comparison of experimental results on untreated reference surfaces and enhanced surfaces “BH” with predictions of Zuber’s and Rohsenow’s correlations]; Section S5. Repeatability of Surface Functionalization [Figure S3. Comparison of boiling performance of multiple specimens of reference (a), type A (b), type B (c) and hydrophobized BH (d) surfaces]; Section S6. Aging of Surfaces During Exposure to Boiling of Water [Figure S4. Boiling performance of individual surfaces during multiple consecutive experiments with water: reference surface (a), type A surface (b), type B surface (c) and hydrophobized BH surface (d)]; Section S7. Boiling Performance Repeatability of BH Surfaces During Boiling of Self-Rewetting Fluids [Figure S5. Boiling performance of the hydrophobized surface BH during the first series of repeated measurements with a self-rewetting fluid (a) and comparison of selected concentrations from the first, the second and the third series of measurements (b)]; Section S8. Aging of Surfaces During Exposure to Boiling of Novec™ 649 [Figure S6. Boiling performance of individual surfaces during multiple consecutive experiments with Novec™ 649: reference surface (a), type A surface (b), hydrophobized surface AH (c), type B surface (d) and hydrophobized surface BH (e)].
