# Supplementary material for: Hydrophilic and Hydrophobic Nanostructured Copper Surfaces for Efficient Pool Boiling Heat Transfer with Water, Water/Butanol Mixtures and Novec 649
Source: Nanomaterials (Basel). 2021 Nov 26;11(12):3216. doi: 10.3390/nano11123216 (PMC8707367; doi:10.3390/nano11123216)
Supplement: Supplementary file 1 [file nanomaterials-1469348-supplementary.pdf › nanomaterials-1469348-supplementary.pdf]

Supplementary Information

Hydrophilic and Hydrophobic Nanostructured Copper Surfaces for Efficient Pool Boiling Heat Transfer with Water, Water/Butanol Mixtures and Novec 649

Matic Može [1,*], Viktor Vajc [2], Matevž Zupančič [1,*] and Iztok Golobič [1]

[1] Faculty of Mechanical Engineering, University of Ljubljana, Aškerčeva 6, 1000 Ljubljana, Slovenia; iztok.golobic@fs.uni-lj.si

[2] Faculty of Mechanical Engineering, Czech Technical University in Prague, Technická 4, 160 00 Prague 6, Czech Republic; viktor.vajc@fs.cvut.cz

* Correspondence: matic.moze@fs.uni-lj.si (M.M.); matevz.zupancic@fs.uni-lj.si (M.Z.); Tel.: +386-1-4771-309 (M.M.; M.Z.)

1. Experimental Setup for Boiling Performance Evaluation

Pool boiling performance of developed surfaces was evaluated using a previously developed experimental setup [1], shown schematically in Fig. S1(a). The setup consists of a glass cylinder with an internal diameter of 60 mm, which serves as the boiling vessel and is clamped between two stainless-steel flanges. The sample, shown in Fig. S1(b), is mounted onto a copper heating block. PEEK bushing, silicon O-rings and epoxy glue were used to seal the edge of the sample. Heat is generated with cartridge heaters inside the heating block and a variable transformer is used to control the heat load. An immersion heater, controlled by another variable transformer, is used to preheat and degas the boiling fluid in the vessel and to maintain the saturation state during the measurements. The produced vapor is led into a water-cooled glass condenser and returned to the vessel.

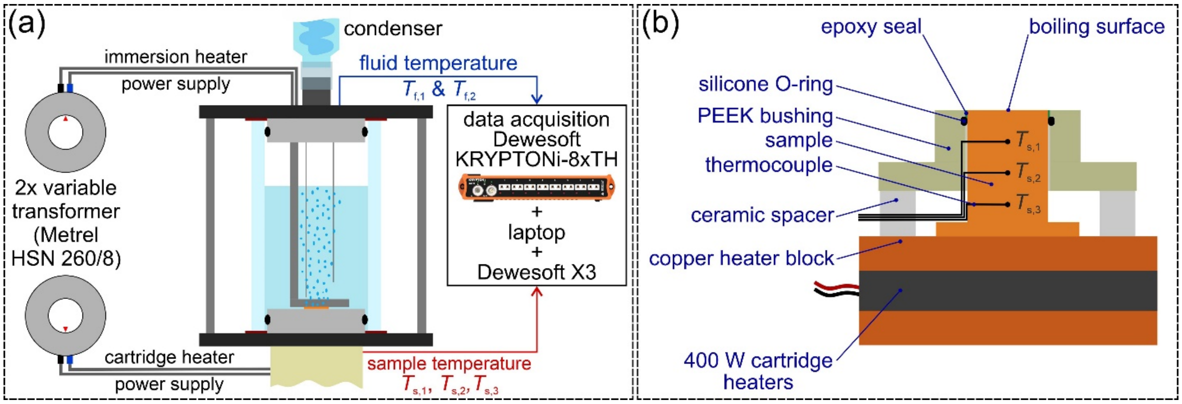

Figure S1. Experimental setup **(a)** and cross-section of the sample on heating assembly **(b)**.

Temperatures inside the sample are measured with three type K thermocouples inside the sample, spaced $\Delta x_1 = 5.0$ mm apart. The thermocouple closest to the boiling surface is positioned $\Delta x_2 = 5.3$ mm from the top of the sample. Two sheathed thermocouples are submerged into the vessel from the top flange and are used to record the temperature of the boiling fluid. All thermocouple signals are collected with a Dewesoft KRYPTONi-8xTH device as raw voltages. Temperature of each cold junction is recorded internally and used to offset the measurements to obtain correct temperature readings. NIST 9th degree polynomial is used to calculate the temperatures based on offset voltages.

2. Data Reduction and Measurement Uncertainty

The following methodology was applied to evaluate pool boiling performance of investigated surfaces. Heat flux $\dot{q}$ was calculated using Fourier's law of conduction. As the

sample is well-insulated and its thermal conductivity is at least three orders of magnitude greater than that of the surrounding materials, only 1D conduction through the sample towards the boiling surface was considered. Based on methodology proposed in [2], linear interpolation was used to calculate the spatial temperature gradient. Using temperature measurements performed with the topmost and bottommost thermocouple, heat flux was evaluated through the following equation:

$$\dot{q} = k\frac{\Delta T}{\Delta x} = k\frac{T_{s,3} - T_{s,1}}{2\Delta x_1} \ , \tag{S1}$$

where $T_{s,3}$ and $T_{s,1}$ are the temperatures measured with the topmost and bottommost thermocouple, respectively, and Δx_1 is the distance between two neighboring thermocouples. Thermal conductivity k was evaluated at the mean temperature in the sample using the following expression:

The latter expression, which uses temperature T in °C and returns the value of thermal conductivity k in W m^{-1} K^{-1}, was established through laser-flash measurements of thermal diffusivity at different temperatures and calculation of the thermal conductivity using temperature-dependent density and specific heat capacity. This was done in order to

$$k(T) = 0.000283T^2 - 0.1646T + 378.07 \ . \tag{S2}$$

avoid calculation mistakes due to overestimated thermal conductivity as advised in [2].

The temperature of the boiling surface T_w is obtained through linear extrapolation from the calculated heat flux and the temperature $T_{s,1}$ measured by the uppermost thermocouple:

$$T_w = T_{s,1} - \frac{\dot{q}\Delta x_2}{k}, \tag{S3}$$

where Δx_2 is the distance between the uppermost thermocouple and the boiling surface. Thermal conductivity is first calculated at $T_{s,1}$ using Eq. (2) to obtain a surface temperature estimate. Average temperature of the upper part of the sample is then calculated as the mean value between the estimated surface temperature and the temperature of the uppermost thermocouple. This average temperature was then used to calculate the best estimate of the mean thermal conductivity of the analyzed section of the sample and to finally extrapolate the surface temperature with improved accuracy. Surface superheat ($T_w - T_{sat}$) is calculated as the difference between the surface temperature and the mean fluid temperature which was measured with two submerged type K sheathed thermocouples. Since all measurements were conducted in the saturated state, temperature of the liquid is equal to saturation temperature T_{sat}.

Heat transfer coefficient h is calculated by dividing the heat flux with the corresponding surface superheat:

$$h = \frac{\dot{q}}{T_{\mathrm{w}} - T_{\mathrm{sat}}}. \tag{S4}$$

Methodology from [2] was used to evaluate the measurement uncertainty of heat flux, superheat and HTC. The following contributing uncertainties were taken into account: (i) the uncertainty of the distance between the thermocouples used to determine the spatial temperature gradient ($u(\Delta x_1)$ = 0.16 mm; evaluated experimentally), (ii) the uncertainty of the distance between the uppermost thermocouple and the boiling surface ($u(\Delta x_2)$ = 0.18 mm; evaluated experimentally, (iii) the uncertainty of the thermocouple temperature measurements ($u(T)$ = 0.19 K at 100 °C and $u(T)$ = 0.30 K at 250 °C; evaluated experimentally) and (iv) the relative uncertainty of thermal conductivity ($u_r(k) \cong 1.5\%$; estimated based on the accuracy of thermal diffusivity measurements).

For boiling of water, the following measurement uncertainties of heat flux, superheat and HTC were evaluated. The combined standard relative uncertainty of the heat flux is the highest at low heat fluxes (~10.4% at 100 kW m^{-2}) and reduces to approx. 2.3% at heat fluxes above 1000 kW m^{-2}. The combined standard uncertainty of surface superheat increases with heat flux from approx. 0.38 K at 100 kW m^{-2} to approx. 1.04 K at 1000 kW m^{-2}. The combined standard relative uncertainty of HTC decreases from approx. 14% at 100 kW m^{-2} to approx. 8.4% at 1000 kW m^{-2}. The exact value of HTC uncertainty depends on boiling performance of individual samples. Since the value of heat transfer parameters during boiling of water/butanol mixtures are similar to those for pure water, the aforementioned uncertainty values are also valid for the experiments with SRFs.

However, heat fluxes and HTCs are approx. one order of magnitude lower for boiling of Novec™ 649 compared with water, resulting in different uncertainties. The combined standard relative uncertainty of heat flux is the highest at low heat fluxes (~17% at 60 kW m^{-2}) and reduces to approx. 6.0% close to the CHF. The combined standard uncertainty of surface superheat increases very slightly with increasing heat flux from approx. 0.37 K at 60 kW m^{-2} to approx. 0.39 K at 180 kW m^{-2}. Finally, the combined standard relative uncertainty of the HTC decreases from approx. 18% at 60 kW m^{-2} to approx. 7.2% at 180 kW m^{-2}, while the exact value is, again, heavily dependent on the performance of an individual surface.

3. Measurement Protocols for Boiling Performance Evaluation

The same measurement protocol was used for experiments with twice-distilled water and Novec™ 649. After mounting the sample and completing the assembly of the measurement setup, the boiling vessel was filled with approx. 200 g of the selected fluid. The immersion heater was then used to bring the fluid's temperature to saturation and 45 min of degassing through vigorous boiling and condensation were performed to eliminate non-condensable gasses entrapped on the boiling surface or in the working fluid. During the degassing, nucleate boiling was also established on the surface to further facilitate the degassing process. Afterwards, the fluid and the surface were cooled down to at least 5 K below saturation to help condense any entrapped vapor. This subcooling process was also used between subsequent experimental runs performed on the same sample. The temperature of the fluid was then again brought to saturation and data acquisition was started.

During each measurement of the boiling curve, the heat flux was slowly but continuously increased by increasing the voltage supplied to the cartridge heaters in the copper heating block. The heat flux change rate was limited to 0.2 kW m^{-2} s^{-1} in the natural convection regime and to 2.5 kW m^{-2} s^{-1} in the nucleate boiling regime. Numerical and experimental validation of this approach was performed as a part of our previous study [1] where it was shown that the quasi-steady-state results obtained in this fashion do not differ from the measurements performed in discrete steady states. When the CHF incipience was achieved, the cartridge heaters were turned off and the sample was left to cool down on its own. Multiple experimental runs were conducted on each sample to evaluate the stability of the surfaces.

Experiments with self-rewetting fluids (SRFs) required a slightly modified approach to account for different concentrations of butanol in water. Concentrations between 1 and 6 wt. % of butanol were tested (1-butanol is soluble in water up to approx. 7 wt. %). To be able to clearly differentiate between the surface aging due to repeated experiments and the effect of SRFs, each surface was first aged through a multitude of boiling runs performed with water until its boiling curve stabilized. Only then were SRF measurements commenced. During these experiments, the SRF concentration was increased by 1 wt. % during each experimental run by adding the appropriate amount of 1-butanol to the known mass of the boiling fluid in the vessel. After the concentration of 6 wt. % was reached, the setup was emptied and all of its interior was thoroughly rinsed several times with pure distilled water. Afterwards, additional series of measurements was conducted by performing another run with pure water and then with SRFs. Since the setup was not completely disassembled during rinsing, a small contamination with 1-butanol residues is possible but was found to be below 0.1 wt. % based on the relation between the saturation temperature of the mixture and its concentration.

4. Comparison of Boiling Performance with Established Models

Boiling performance of untreated surfaces was compared with the most common models found in the literature to confirm the validity of the reference values and their comparability with other studies. This evaluation was performed both for pure water and Novec™ 649. The thermophysical properties of water (REFPROP 10.0) and Novec™ 649 [3–5] taken into account in the calculations are listed in Table S1. The values for water were obtained for saturation temperature at atmospheric pressure. The values for Novec™ 649 were obtained for saturation temperature at a pressure of 1.15 atm with the exception of thermal conductivity and dynamic viscosity, which could only be sourced at 25 °C.

Table S1. Thermophysical properties of water and Novec™ 649.

Property / Fluid	ρ_L (kg m-3)	ρ_G (kg m-3)	σ (mN m-1)	μ_L (mPa s)	$c_{p,L}$ (J kg k-1)	Δh_{LG} (kJ kg-1)	k_L (W m-1 K-1	Pr (/)
Water	958	0.598	58.9	0.282	4216	2257	0.677	1.76
Novec™ 649	1505	12.2	10.8	0.676	1121	86.9	0.059	12.8

Zuber's correlation [6,7] was used to estimate the critical heat flux value on the reference surfaces. The correlation primarily takes into account working fluid's thermophysical properties to estimate at which heat flux hydrodynamic instability will inhibit the supply of fresh liquid to the surface and results and creates an insulative vapor film over the surface. The correlation is written in the following form:

$$\dot{q}_{\mathrm{CHF}} = K\sqrt{\rho_{\mathrm{G}}}h_{\mathrm{LG}}[\sigma g(\rho_{\mathrm{L}} - \rho_{\mathrm{G}})]^{1/4}\,. \quad \text{(S5)}$$

The recommended value of the constant K is 0.131. This yields an expected critical heat flux (CHF) value of 1109 kW m-2 and 141 kW m-2 for water and Novec™ 649, respectively. Within this study CHF values between 858 kW m-2 and 1208 kW m-2 were recorded experimentally on untreated surfaces with water. The predicted CHF value falls in this range. Furthermore, the observed CHF values also match those summarized in a comparative study by Može et al. [2]. The experimentally observed CHF value on an untreated surface with Novec™ 649 (201 kW m-2) is noticeably higher than the value predicted through Zuber's model. Nevertheless, it matches experimental observations by Ghaffari et al. [8], who measured the CHF of Novec™ 649 to be 195 kW m-2 on bare copper surface under pool boiling conditions.

The prediction of Zuber's correlation for boiling of water is shown in Figure S2 and compared both with experimental CHF value observed on untreated reference surface and enhanced surfaces "BH".

Heat transfer coefficient can be estimated using Rohsenow's correlation [9], which connects the value of surface superheat and heat flux within the nucleate boiling regime:

$$\frac{c_{p,\mathrm{L}}\Delta T_{\mathrm{sat}}}{\Delta h_{\mathrm{LG}}} = C_{\mathrm{sf}}\left\{\frac{\dot{q}}{\mu_{\mathrm{L}}\Delta h_{\mathrm{LG}}}\sqrt{\frac{\sigma}{g(\rho_{\mathrm{L}}-\rho_{\mathrm{G}})}}\right\}^{1/3}\left[\frac{c_{p,\mathrm{L}}\mu_{\mathrm{L}}}{k_{\mathrm{L}}}\right]^{n}. \quad \text{(S6)}$$

The value of the C_{sf} parameter depends on the combination of the fluid and boiling surface material (and condition). It is suggested to fix the exponent n at 1 for water and 1.7 for all other fluids.

Figure S2 shows a comparison of boiling curves recorded with water on four different untreated reference surfaces. Prediction of Rohsenow's correlation is shown for the best fit of the experimental data with a C_{sf} value of 0.0157 ($R^2 = 0.917$). Furthermore, Rohsenow's correlation fitted onto experimental data for three copies of type "BH" enhanced surface is also shown ($C_{\mathrm{sf}} = 0.0098$; $R^2 = 0.998$). As it is evident from Fig. S2, the recorded CHF values match those predicted by the Zuber's correlation and the boiling curves within the nucleate boiling regime are described well by Rohsenow's correlation. However, enhanced surfaces exceed the CHF value predicted by Zuber's correlation and require a significantly different C_{sf} parameter value in Rohsenow's correlation.

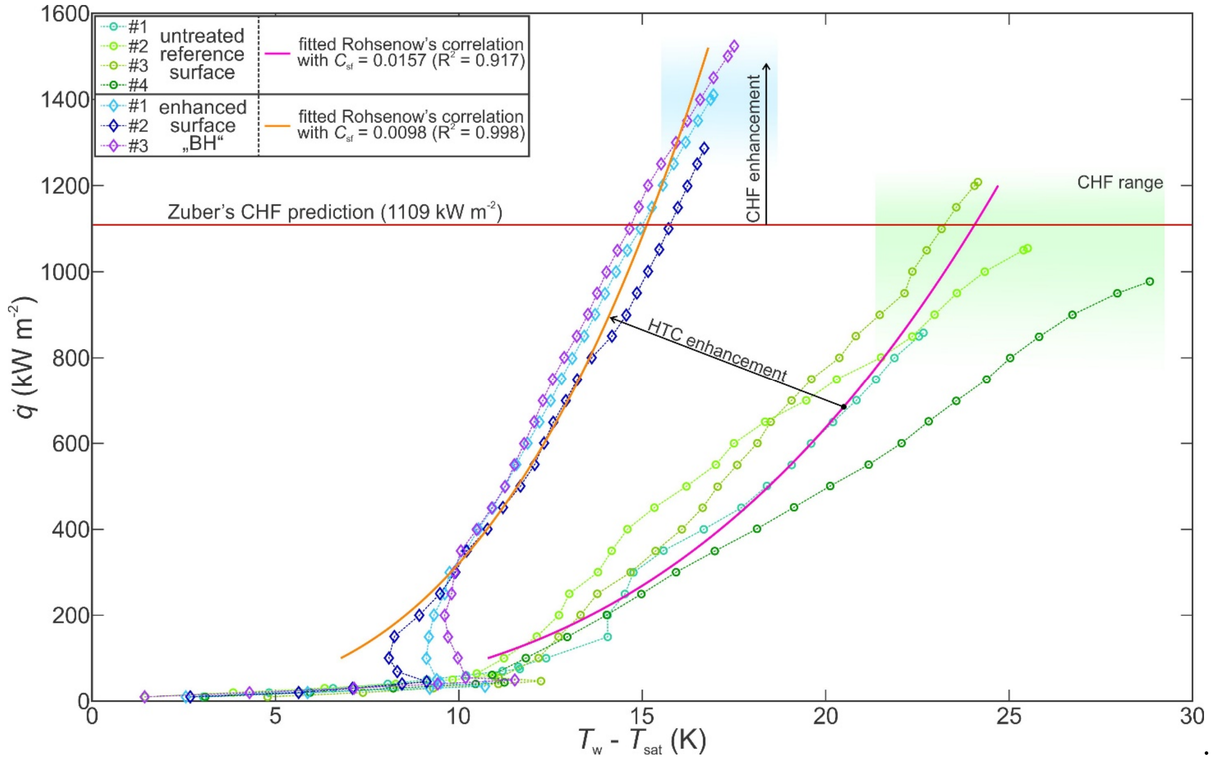

Figure S2. Comparison of experimental results on untreated reference surfaces and enhanced surfaces "BH" with predictions of Zuber's and Rohsenow's correlations.

5. Repeatability of Surface Functionalization

To evaluate the repeatability of the surface functionalization process, multiple copies of selected surface were prepared, and their boiling performance was evaluated under the same conditions. In addition to the reference surface, the repeatability tests were performed on surfaces with most favorable heat transfer characteristics, which were identified in main text subsection 3.2.1 (i.e., surfaces A, B and BH). The results of this evaluation are shown in Figure S3.

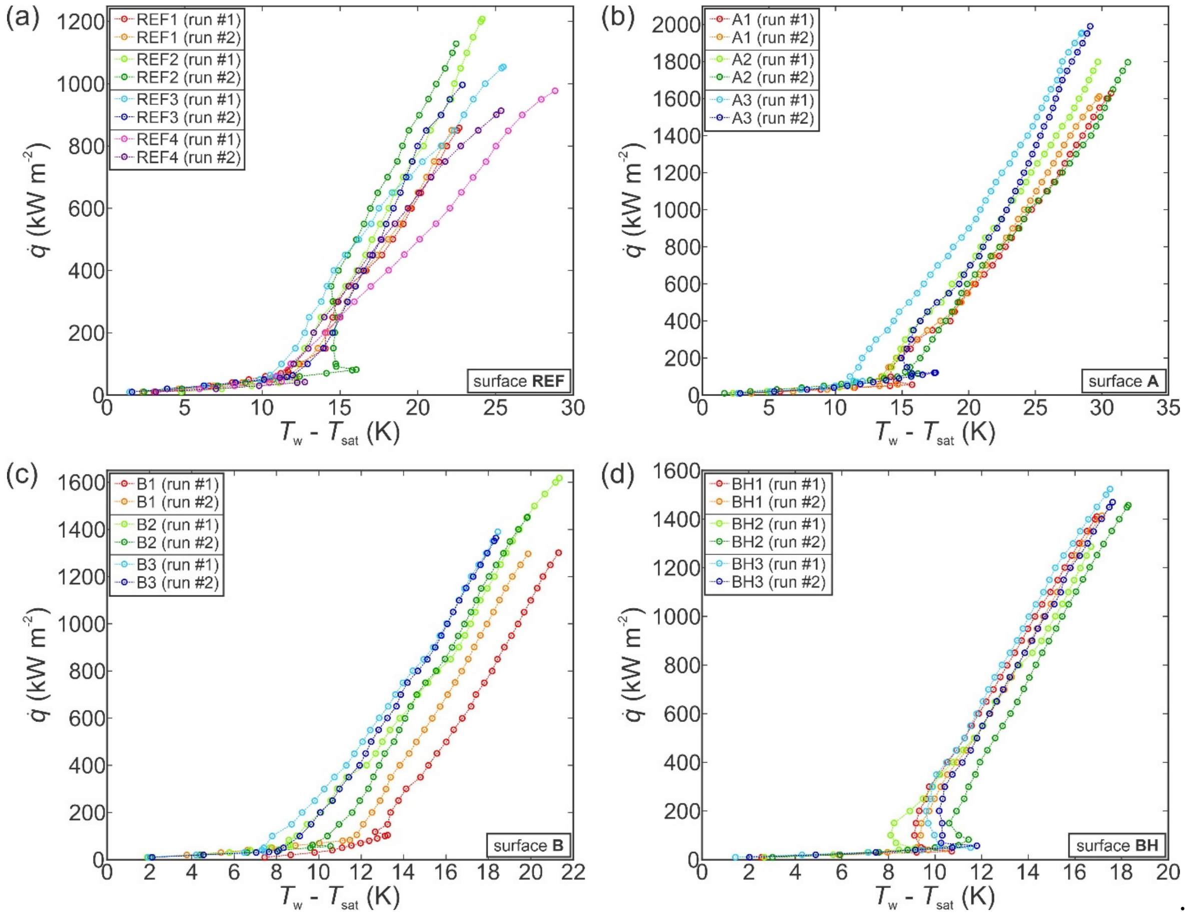

Figure S3. Comparison of boiling performance of multiple specimens of reference (a), type A (b), type B (c) and hydrophobized BH (d) surfaces.

Firstly, some scatter was observed between the performance of four reference surfaces. This is not entirely unexpected as a recent study [2] showed that while some deviations between the reported CHF values stem from different data reduction methods and measurement uncertainty, it does not fully explain the entirety of the scatter. Additional reasons such as (uncontrolled) adsorption of volatile organic compounds onto the surface [10] or the inherent randomness of the boiling process can be responsible for the observed deviations. The deviations were much lower on the type A functionalized surfaces with CHF values within 20% of one another and surface superheats at CHF deviations for less than 5 K. Slightly larger deviations in terms of surface superheat and the point of nucleate boiling onset were observed for type B surfaces as evident from Figure S3c. Finally, hydrophobized surfaces BH exhibited the most favorable fabrication repeatability with almost negligible differences between the boiling curves observed on three separate surfaces, see Figure S3d. Overall, the fabrication repeatability is deemed as satisfactory since multiple copies of all investigated surfaces exhibited comparable boiling performance.

6. Aging of Surfaces During Exposure to Boiling of Water

In realistic applications, boiling surfaces would be exposed to the boiling process for prolonged periods of time. However, their aging performance is often neglected [11]. One method of evaluating the degradation stemming from exposure to boiling is to expose the surface to multiple subsequent incipiencies of the critical heat flux where the temperature of the surfaces increases substantially and affects surface chemistry and morphology [12]. Therefore, selected surfaces (the same selection as in subsection S1 was used) were exposed to multiple subsequent boiling curve measurements with each one carried out until

the CHF incipience and subsequent transition towards film boiling. The results of surface aging evaluation are compared in Fig. S4.

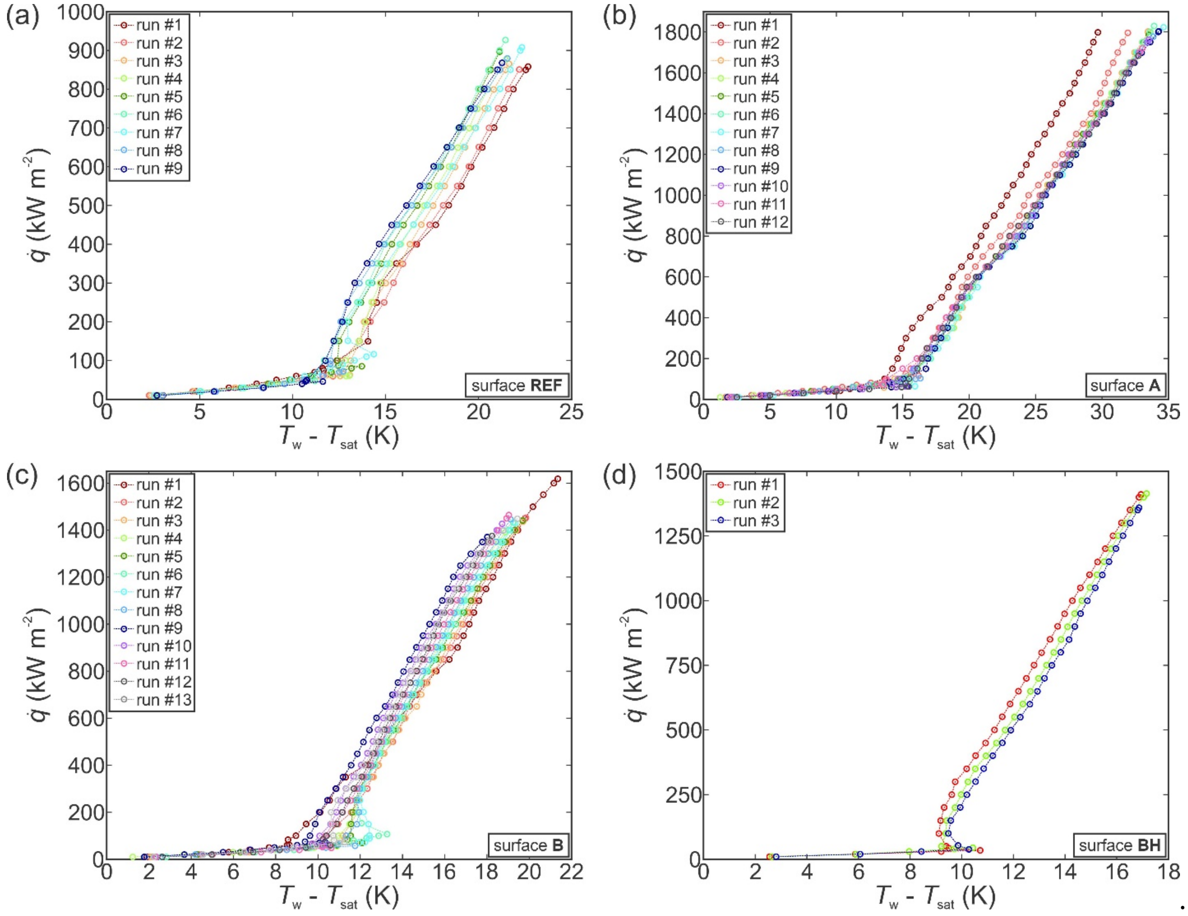

Figure S4. Boiling performance of individual surfaces during multiple consecutive experiments with water: reference surface (a), type A surface (b), type B surface (c) and hydrophobized BH surface (d).

Nine consecutive experimental runs on the untreated surface, shown in Fig. S4(a), resulted in both a slight increase of the CHF value and a slight shift of the boiling curve towards lower superheats. Overall, the performance stabilized after approximately five runs. Fig. S4(b) shows aging evaluation results obtained on the type A surface. Here, a noticeable shift of the boiling curve occurred after the first experiment, followed by a smaller shift after the second run. The boiling performance and the position of the boiling curve stabilized thereafter, and no deviations were observed in the nine following experimental runs. This can be attributed to the CuO surface structures which seem to be inert when exposed to elevated temperatures in the presence of pure water – no additional oxidation processes seem to have taken place. Repeatable boiling performance of type A surfaces was also reported by Raman and McCarthy [13].

On the contrary, type B surface shown in Fig. S4(c) exhibited a gradual shift of the boiling curve within the thirteen experimental runs followed by stabilization of heat transfer parameters. The CHF value decreased by about 10% while the surface superheat also decreased. Stabilized performance was achieved after approximately ten experiments. Since the expected oxides and hydroxides did not form during the oxidation treatment on the type B surface, the surface was likely more prone to oxidation when exposed to boiling water for a prolonged amount of time. However, the change in boiling performance is overall quite small and stable performance was observed after the aging period. Lastly, hydrophobized surface BH was also tested. This surface exhibited by far the most stable boiling performance as evident from Fig. S4(d). Here, the experiments were discontinued

after three runs due to the stable performance and unchanging position of the boiling curve including a constant CHF value. Stable performance of surfaces coated with a fluorinated silane was reported previously [1,14]. The thin layer of the hydrophobic agent seems to protect the surface from oxidation processes even when exposed to boiling for multiple hours.

7. Boiling Performance Repeatability of BH Surfaces During Boiling of Self-Rewetting Fluids

To further verify the observed trends on the hydrophobized surface BH, which was the only one providing stable performance with BuOH mixtures, another copy of it was prepared and tested with 2, 4 and 6 wt. % of BuOH. The results in Fig. S5(a) indicate that no degradation seems to take place during the first series of experimental runs. The experiments were then repeated with water and BuOH concentrations of 2 and 6 wt. %; these results are shown as the second series in Fig. S5(b). Performance with water remains the same and boiling curves for 2 and 6 wt. % BuOH mixtures also match those from the first series of measurements well. From this, we can conclude that the second BH surface did not degrade in contact with boiling water/butanol mixtures, and it appears that the fluorinated silane coating at least temporarily protects the surface against reaction with 1-butanol.

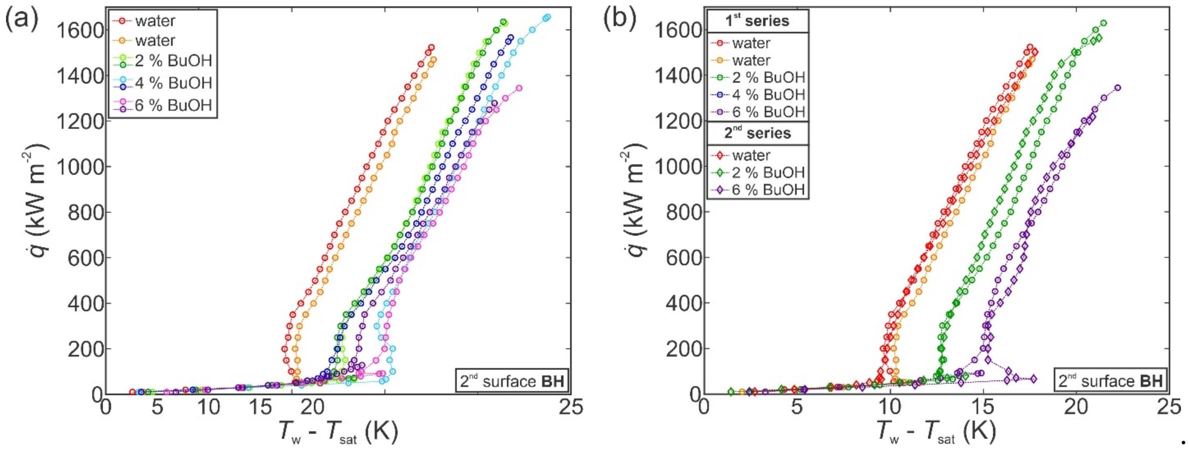

Figure S5. Boiling performance of the hydrophobized surface BH during the first series of repeated measurements with a self-rewetting fluid (a) and comparison of selected concentrations from the first, the second and the third series of measurements (b).

8. Aging of Surfaces During Exposure to Boiling of Novec™ 649

Aging of surfaces when exposed to boiling of Novec™ 649 was evaluated the same way as for water experiments. Three to five consecutive CHF onsets were achieved on each of the tested surface and the resulting shift in boiling curve was analyzed. Surface aging for individual functionalized surfaces is shown in Fig. S6 and compared with the aging of the untreated surface.

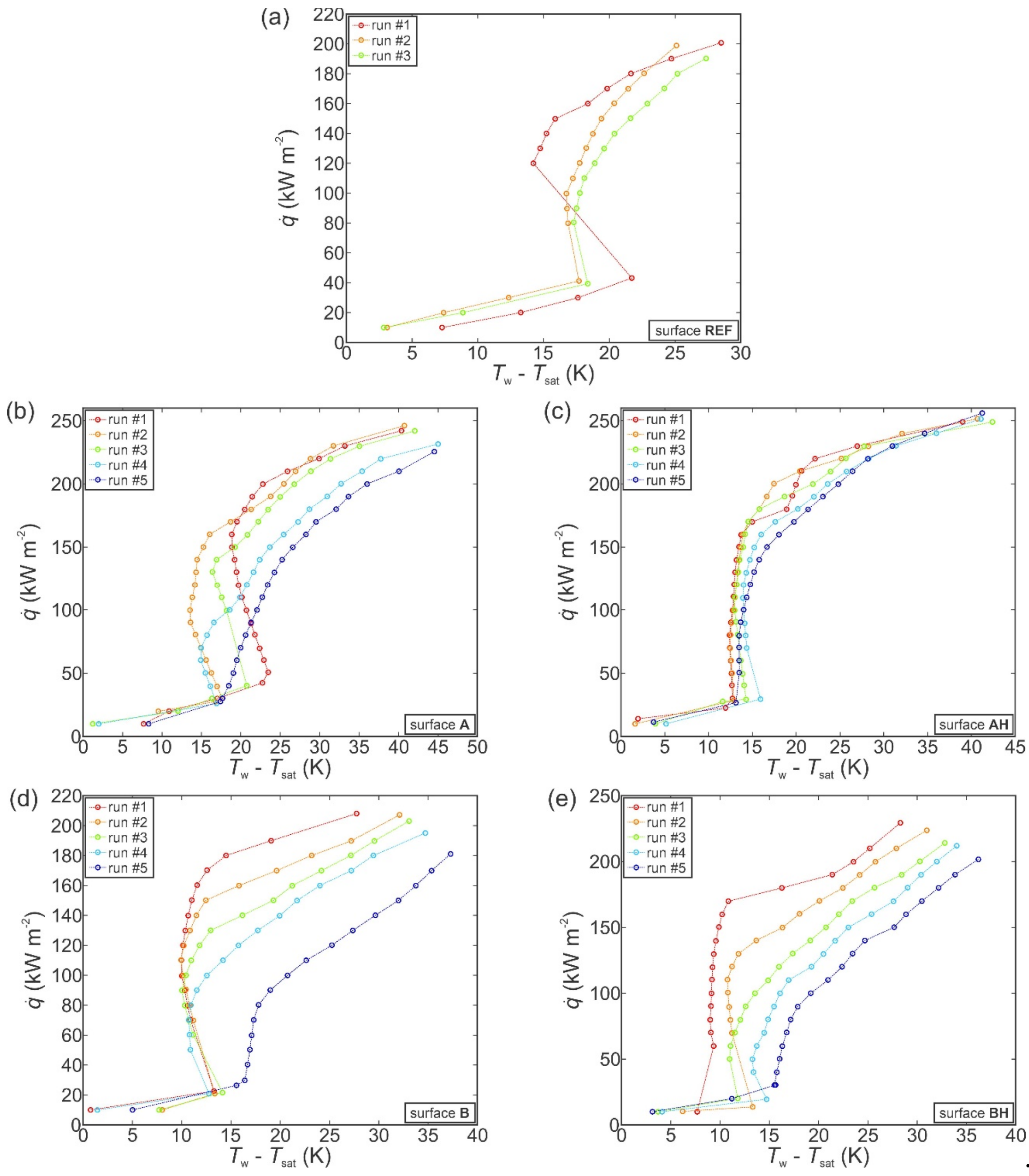

Figure S6. Boiling performance of individual surfaces during multiple consecutive experiments with Novec™ 649: reference surface (a), type A surface (b), hydrophobized surface AH (c), type B surface (d) and hydrophobized surface BH (e).

The aging evaluation suggests that exposure to boiling of Novec™ 649 degrades the boiling heat transfer performance. Figure S6(a) shows that the CHF values remained nearly constant on the reference surface, but the surface superheat increased with every run. Coversely, the superheat required for ONB lowered. This is also true for the type A surface, see Fig. S6(b), where the heat transfer initially improved with a shift of the boiling curve towards lower superheat values (run #2) but then degraded with a gradual shift towards higher superheat values and with approx. 10% decrease in CHF. Fig. S6(d) shows only a minor shift of the boiling curve towards higher superheats observed at ~200 kW m^{-2} and also indicates that the hydrophobic coating of the AH sample seems to have protected the surface against degradation, resembling the resistance of the BH surface against degradation during boiling of water/butanol mixtures.

Major degradation of the heat transfer parameters was observed on both the non-hydrophobized surface BH and its hydrophobized variant (Fig. S6(d) and Fig. S6(e),

respectively). In both cases the boiling curve continuously shifted towards higher superheat values with every subsequent measurement. Similarly, the CHF value gradually decreased by approx. 13% (surface B) and 12% (surface BH). Here, the fluorinated silane coating failed to protect the type B surface from chemical reaction with the used dielectric fluid. Since the opposite was observed on the hydrophobized surface AH, coating itself probably did not degrade, but rather detached from the surface due to its poor adhesion to the substrate. The exact mechanism of through which copper surfaces degrade when exposed to boiling of Novec™ 649 requires further investigation, which is outside of the scope of this study. Also, attention should be paid to the stability, aging and suitable functionalization of surfaces aimed for boiling of Novec™ 649.

References

1. Može, M.; Vajc, V.; Zupančič, M.; Šulc, R.; Golobič, I. Pool boiling performance of water and self-rewetting fluids on hybrid functionalized aluminum surfaces. *Processes* **2021**, *9*, doi:10.3390/pr9061058.
2. Može, M.; Zupančič, M.; Golobič, I. Investigation of the scatter in reported pool boiling CHF measurements including analysis of heat flux and measurement uncertainty evaluation methodology. *Appl. Therm. Eng.* **2020**, *169*, 114938, doi:10.1016/j.applthermaleng.2020.114938.
3. Forrest, E.C.; Hu, L.-W.; Buongiorno, J.; McKrell, T. Pool Boiling Performance of Novec 649 Engineered Fluid. *ECI Int. Conf. Boil. Heat Transf. Florianop.* **2009**, *1*, 1–7.
4. Tuma, P.E. Fluoroketone C2F5C(O)CF(CF3)2 as a heat transfer fluid for passive and pumped 2-phase applications. *Annu. IEEE Semicond. Therm. Meas. Manag. Symp.* **2008**, 173–179, doi:10.1109/STHERM.2008.4509386.
5. Bruder, M.; Sembach, L.; Krumova, V.; Sattelmayer, T. Local data of heat flux, wall temperature and the void phase along the boiling curve during vertical subcooled flow boiling of refrigerant Novec 649 at a copper wall. *Data Br.* **2018**, *21*, 1415–1429, doi:10.1016/j.dib.2018.10.138.
6. Zuber, N. Hydrodynamic Aspects of Boiling Heat Transfer: PhD Thesis, UCLA, Los Angeles, USA, 1959.
7. Zuber, N. On the stability of boiling heat transfer. *Trans. Am. Soc. Mech. Eng.* **1958**, *80*, 711–720.
8. Ghaffari, O.; Grenier, F.; Morissette, J.-F.; Bolduc, M.; Jasmin, S.; Sylvestre, J. Pool Boiling Experiment of Dielectric Liquids and Numerical Study for Cooling a Microprocessor. In Proceedings of the 2019 18th IEEE Intersociety Conference on Thermal and Thermomechanical Phenomena in Electronic Systems (ITherm); IEEE, 2019; pp. 540–545.
9. Rohsenow, W.M. A method of correlating heat transfer data for surface boiling of liquids. *Trans. ASME* **1952**, *74*, 969–976.
10. Song, Y.; Zhang, L.; Liu, Z.; Preston, D.J.; Wang, E.N. Effects of airborne hydrocarbon adsorption on pool boiling heat transfer. *Appl. Phys. Lett.* **2020**, *116*, doi:10.1063/5.0012839.
11. Može, M. Effect of boiling-induced aging on pool boiling heat transfer performance of untreated and laser-textured copper surfaces. *Appl. Therm. Eng.* **2020**, *181*, 116025, doi:10.1016/j.applthermaleng.2020.116025.
12. Može, M.; Zupančič, M.; Hočevar, M.; Golobič, I.; Gregorčič, P. Surface chemistry and morphology transition induced by critical heat flux incipience on laser-textured copper surfaces. *Appl. Surf. Sci.* **2019**, *490*, 220–230, doi:10.1016/j.apsusc.2019.06.068.
13. Rahman, M.M.; McCarthy, M. Boiling Enhancement on Nanostructured Surfaces with Engineered Variations in Wettability and Thermal Conductivity. *Heat Transf. Eng.* **2017**, *38*, 1285–1295, doi:10.1080/01457632.2016.1242961.
14. Može, M.; Senegačnik, M.; Gregorčič, P.; Hočevar, M.; Zupančič, M.; Golobič, I. Laser-Engineered Microcavity Surfaces with a Nanoscale Superhydrophobic Coating for Extreme Boiling Performance. *ACS Appl. Mater. Interfaces* **2020**, *12*, 24419–24431, doi:10.1021/acsami.0c01594.

•

.
